# Exploring associations between water, sanitation, and anemia through 47 nationally representative demographic and health surveys

**DOI:** 10.1111/nyas.14109

**Published:** 2019-06-24

**Authors:** Monica T. Kothari, Amanda Coile, Arja Huestis, Tom Pullum, Dean Garrett, Cyril Engmann

**Affiliations:** ^1^ Maternal, Newborn, Child Health and Nutrition (MNCHN), PATH Washington DC; ^2^ ICF, Demographic Health Surveys Rockville Maryland; ^3^ Departments of Pediatrics and Global Health, Schools of Medicine and Public Health University of Washington Seattle Washington

**Keywords:** anemia, water, sanitation, demographic health survey, nutrition

## Abstract

Globally, no countries are on track to achieve the adopted global nutrition targets set for anemia in 2025. Given the linkages between water, sanitation, and hygiene (WASH) and nutrition, this secondary data analysis explores potential associations with anemia. Forty‐seven demographic and health surveys were used to explore the association between unimproved water and sanitation and anemia in women and children with adjusted odds ratios (ORs) calculated by country and cumulatively. In over 60% of countries, children with off‐premises water access had significantly increased odds of anemia. In over a quarter of countries, children exposed to surface water had higher odds of anemia. In Burundi, children were 1.65 times more likely to be anemic when reported to be living in households using surface water. However, in India, a protective effect was noted (adjusted OR: 0.70, *P* < 0.001) for surface water. In 60% and 65% of countries, women and children exposed to an open sanitation facility had higher odds of being anemic, respectively. There is evidence of an association between selected water and sanitation indicators and anemia. Promoting policies, practices and research that strengthen access to improved WASH should be considered for reducing anemia prevalence alongside standard nutrition interventions.

## Introduction and rationale

Globally, no countries are on track to achieve the adopted global nutrition targets set for anemia in 2025.^1^ Progress in addressing anemia has been slow, and anemia prevalence has even risen slightly in women of reproductive age (32.8% in 2016 compared with 31.6% in 2000).^1^ The morbidity, mortality, and economic ramifications associated with anemia are numerous, in some cases irreversible, and have a lifelong impact.^2^ Children under the age of five, adolescent girls, and pregnant women are the most vulnerable population to suffer from this condition. About 96.07 million children and 202 million women are currently affected by anemia.^3^, ^4^, ^5^ In children, anemia may contribute to poor growth, stunting, cognitive delays, and increased susceptibility to infections.^6^ In women, studies have highlighted an increased risk of anemia during pregnancy with increasing parity.^7^, ^8^ Anemia has been known to increase the risk of adverse pregnancy outcomes, including low birth weight and small for gestational age infants, and also impair work productivity.^9^

Anemia onset can be caused by a myriad of factors. These include genetic factors, such as thalassemia and sickle cell disorders; diseases, such as cancer; micronutrient deficiencies; infections, such as malaria, tuberculosis, and HIV/AIDS; and helminthic infestations (e.g., hookworm and schistosomiasis).^3^, ^6^, ^10^ The prevalence and etiology of anemia vary by region. Iron deficiency until recently was thought to be the most common cause of anemia in low‐ and middle‐income countries.^11^ In 2015, the World Health Organization (WHO) estimated that iron deficiency anemia (IDA) accounts for roughly 50% of cases in women and 42% in children.^11^ As indicated, nutrition interventions alone have been unsuccessful in normalizing hemoglobin (Hb) levels. For instance, iron‐focused interventions have only reportedly resolved 50% of anemia in children.^7^, ^12^, ^13^, ^14^ This conveys the importance of moving beyond a nutrition‐specific^1^ approach alone, to one that considers the broader context in which anemia arises.

Water, sanitation, and hygiene (WASH) practices, in addition to safe water provision, a supply of adequate sanitation, and proper hygiene education can significantly reduce illness and death, improve child health outcomes, and socioeconomic development.^11^, ^15^, ^16^, ^17^ According to a 2015 WHO/UNICEF report, approximately 30% of the world's population did not have access to safely managed drinking water (located on‐premises, available when needed, and free of contamination) and less than 40% of the world's population used a safely managed sanitation service; “that is, excreta safely disposed of *in situ* or treated off‐site.”^11^ Thus, improvements in WASH have been identified as a key sustainable development goal.^18^, ^19^

There is increasing appreciation for identifying pathways by which poor WASH impacts nutrition.^20^, ^21^, ^22^, ^23^ An analysis of demographic and health surveys (DHS) data from 1986 to 2007 found strong protective effects of improved water and sanitation on child health, with access to improved sanitation and water associated with lower odds of child diarrhea, as well as mild or severe stunting.^17^ About 50% of the underweight or malnutrition burden in children under five can be attributed to recurrent diarrhea or intestinal helminth infections that result from poor WASH.^24^ In addition, a report from the World Bank, which used predictive modeling, estimated that 54% of child height prediction can be explained by open defecation alone, hence attributable to sanitation.^25^ Specific to anemia, a recent meta‐analysis of DHS and multiple indicator cluster surveys data showed that community‐level sanitation access was associated with lower odds of any anemia in children, as well as moderate or severe anemia, regardless of individual household sanitation access.^16^

Existing literature shows relevant progress in understanding the linkages between WASH and nutrition. Furthermore, in multisectoral planning efforts, WASH is listed as one of the key nutrition‐sensitive^2^ interventions recommended to improve child nutrition.^26^ To further strengthen this evidence, it is essential to evaluate specific nutrition indicators, such as anemia, in the context of WASH. To this scope, this paper builds on the current evidence and will provide analysis from 47 nationally representative DHS to facilitate a dialogue among key nutrition‐specific and nutrition‐sensitive stakeholders and policymakers on the inclusion of WASH in the multisectoral planning for scaling up nutrition.

## Materials and methodology

### Data

This report uses cross‐sectional data from 47 nationally representative DHS completed between 2006 and 2017, including anemia testing data (see Annex A, online only, for the full list). Only the most recent country survey was used in this analysis to ensure comparability of country‐specific results as the data collection protocols and training for anemia testing has strengthened with time. Only one survey per country was included (the most recent one) to avoid giving more weight toward countries that have multiple surveys in the cumulative estimates. All data collected by DHS are Institutional Review Board (IRB) certified^27^ and this secondary analysis received an exemption from further IRB approval by the PATH Research Determination Committee.

Eligible children aged 6−59 months and women aged 15−49 years are identified for anemia testing. Testing is conducted at the household level, by measuring Hb level in the capillary blood samples collected from a finger or heel prick for children and from finger prick in adults. Individuals eligible for anemia testing and the parents/guardians of eligible children are advised about the objectives, potential risks, voluntary participation in testing, and confidentiality of the anemia testing procedures, as part of the DHS informed consent process. Parents or guardians of never‐married adolescents aged 15−17 are asked for consent to test each adolescent before consent of the adolescent is sought. After obtaining informed consent, a finger (or a heel in the case of children less than 12 months old, or those with small fingers) is cleaned with a swab, impregnated with 70% isopropyl alcohol, allowed to air dry, and pricked with a disposable self‐retracting lancet. The first two blood drops are wiped away; the third drop is collected with a microcuvette for measurement of the Hb level using the HemoCue® Hb 201^+^ analyzer (www.hemocue.com) and results are provided to participants immediately. A standard biomarker questionnaire is used to record Hb for children and women, which are used to categorize the degree of anemia. The any anemia cutoff (Hb < 11.0 g/dL for children and pregnant women; Hb < 12.0 g/dL for nonpregnant women) as recommended by WHO and used by DHS for classifying anemia was used in the study, see Annex A (online only).^28^ DHS adjusts its anemia data for altitude, place of residence, and smoking for adults.

Information on household WASH variables is collected as part of the DHS household questionnaire. The household questionnaire is administered to the head of household selected for the survey based on two‐stage population proportionate to probability sampling.^29^ This questionnaire includes information about characteristics of the household's dwelling unit and characteristics of usual residents and visitors, such as age, sex, residence, education level/maternal education, wealth index, and maternity status. The information about the eligible women and children for anemia testing is also identified in this questionnaire. The eligible women are then interviewed using an individual woman's questionnaire. This questionnaire includes detailed information about the woman herself and about each of her births. Information on iron supplementation and deworming for children and women is also recorded in this questionnaire.

The DHS minimize nonsampling, coverage, and data processing errors by extensively training data collectors/health investigators on the standard internationally recommended protocols and data confidentiality.^29^

Missing data are flagged in the DHS data files and excluded from the denominators. The DHS flagged missing data are excluded from the current analysis. In this analysis, household WASH data are analyzed for children and women as individual units to explore associations with anemia. WASH variables including the type of sanitation facility, the shared status of toilet facility, and the main source and access to drinking water are categorized according to definitions recommended by the WHO/UNICEF Joint Monitoring Programme (JMP) for Water Supply and Sanitation.^30^ Box 1 presents the categorization of the type of sanitation facility and the main source of drinking water variables according to the JMP categorization. Further, the shared status of toilet facility was categorized as not shared or shared (including shared with another household or public toilet) and time to obtain water was categorized as on‐premises or not on‐premises (including all other times) to simplify the analysis. For the analysis, WASH variables were recategorized to calculate composite variables that accounted for the slight variations in questions across countries, such as a missing category for open sanitation facility in a middle‐income country. Footnotes in the results tables indicate any deviations from the standard denominators.

**Box 1. Categories of improved and unimproved sanitation facilities and improved and unimproved water sources constructed using information from DHS household questionnaire (WHO/UNICEF 2017; JMP classification)**

**Table:** 

**Sanitation**		
Improved	Unimproved	Open defecation
Flush or pour‐flush to: ^○^ Piped sewer system ^○^ Septic tank ^○^ Pit latrine Ventilation improved pit (VIP) latrine Pit latrine with slab Composting toilet Latrine with manual flush Ecosan	Flush or pour‐flush to elsewhere (i.e., not to piped sewer system, septic tank, or pit latrine) Pit latrine without slab or open pit Bucket/pan toilet Hanging toilet or hanging latrine Shared or public facilities of any type Dry toilet Other	No facilities Yard Bush Field or forest
**Drinking water**		
**Improved**	**Unimproved**	**Surface water**
Piped water into dwelling, yard, or plot Public tap or standpipe Tube well or borehole Protected spring Protected dug well Rainwater collection	Unprotected dug well Unprotected spring Cart with small tank or drum Tanker truck Bottled water Other	River Dam Lake Pond Stream Canal Irrigation channel

## Analysis plan

All countries included in the analysis account for the sampling design and sampling units *a priori*. For this report, initial descriptive analysis was run for all countries included to show: the distribution of any anemia, socioeconomic factors, iron supplementation, deworming, and water and sanitation characteristics among children and women. To provide further context of the chosen countries, we further summarized household‐level WASH indicators using the DHS online portal (http://www.statcompiler.com/).

We reported on the distribution of anemia by selected background characteristics and water and sanitation indicators, using cross‐tabulations. For ordinal variables, the category with the highest prevalence of anemia is reported when describing patterns. To report patterns for the categorical variables, we either (1) present a trend across the variables (e.g., decreasing prevalence by category) or (2) report the category with the highest prevalence if there was no noticeable trend or the categories were not hierarchical. Countries that had a sample size for a variable less than 25 or that did not include information about a category were removed from the analysis of the association of the specific variable with anemia. Unadjusted odds ratios (ORs) for child sex and deworming were also run to assess the significance of these key factors.

Univariate and minimally adjusted (age and sex for children and age for women) analysis was conducted to explore the association between the main exposures of interest (water and sanitation indicators) and anemia. We adopted the standard *P*‐values of less than 0.05 to denote statistical significance. Forest plots were created to illustrate the difference in the direction and strength of associations between water and sanitation variables for all countries using the minimally adjusted associations. The same *P*‐value cutoff was used to note the variation between the countries. The cumulative ORs are not expected to be subject to selection bias, recall bias, or publication bias as these are expected to become negligible at the comparison stage as all surveys have the same data collection process and are weighted to adjust for sampling design and other key factors prior to analysis.^31^

Multivariate analysis was run for four countries that represented different regions of the world and that showed strong ORs in the univariate analysis across two or more of the water and sanitation indicators. This was necessary given the aim of the study is to further understand the potential associations between poor water and sanitation and anemia and not the association of other factors potentially affecting anemia. Burundi, Guinea, India, and Senegal met analytical requirements for multivariate analysis. In the West Africa region, we present results from Senegal in the multivariate analysis as it qualified our selection criteria and had a larger sample size and more recent survey.

In the multivariate analysis, run separately for women and children, to ensure enough samples sizes across categories when adding additional factors into the regression model, the main exposures of interests were recategorized as follows:

- Water source: improved and on‐premises (0), unimproved or not on premises (1), and surface (2).
- Sanitation: improved and not shared (0), and unimproved or shared (1).

The base model for the regression analysis in both women and children assessed the effect of the above‐mentioned water and sanitation categories on anemia. The adjusted regression models included covariates that are not necessarily on the causal pathway, are significantly associated with anemia in the univariate analysis, could be confounders, and have a sufficient sample size.

## Results

The prevalence of any anemia is 55.1% in children (47 countries, *n* = 385,541) and 38.1% in women (countries: 45, *n* = 1,049,827) based on cross‐sectional data from DHS surveys conducted between 2006 and 2017. In over 95% of countries, the proportion of children who are anemic is higher than that of women, and the direction of this difference stays consistent across all levels of anemia except for “mild anemia,” where women show a higher prevalence (Annex A, online only).

### Anemia prevalence by sociodemographic characteristics

Any anemia status for children and women was disaggregated by standard sociodemographic variables including age, gender (children only), residence, mother's/women's education level, wealth index, and maternity status (women only). Deworming and iron supplementation variables were also included. Table 1 presents a summary and overall directional trend of anemia prevalence among the variable categories.

**Table 1:** Weighted averages of any anemia prevalence and pattern across countries in anemia prevalence among children and women by background characteristics, DHS surveys (2006−2017)

Variable	Categories	Weighted average prevalence of anemia^a^	Percentage of countries with the highest prevalence of anemia by category	Overall prevalence pattern and select associations^b^
**Children, 6–59 months of age**		*n* = 385,541	*n* = 47	
Child age	6−11	71.1	70.2	In 70% of countries, prevalence decreases by age.
	12−23	67.1	29.8	
	24−59	47.8	0.0	
Child sex	Boys	56.1	80.9	In 81% of countries, prevalence is higher among boys. In 11 of 47 countries, the odds of anemia are significantly greater for boys, with an overall effect of 1.05 (*P* < 0.001).
	Girls	54.0	19.1	
Residence	Urban	51.1	17.0	In 83% of countries, prevalence is higher in rural areas.
	Rural	57.0	83.0	
Mother's education^d^	None	60.0	60.9	In 50% of countries, prevalence decreases by increasing maternal education level.
	Primary	55.5	19.6	
	Secondary	50.9	13.0	
	Higher	41.3	6.5	
Wealth quintile	Lowest	59.9	66.0	In 37% of countries, prevalence decreases by increasing wealth index.
	Second	57.5	8.5	
	Middle	55.9	17.0	
	Fourth	52.4	4.3	
	Highest	46.4	4.3	
Iron supplementation^d^	No	56.7	65.9	In 66% of countries, prevalence is higher among children not taking iron supplements.
	Yes	55.9	35.0	
Deworming^d^	No	59.8	95.3	In 95% of countries, prevalence is higher among those reporting not taking deworming medication. In 27 of 47 countries, the odds of anemia were significantly lower for children taking deworming medication.
	Yes	53.2	4.7	
**Women, 15–49 years old**		*n* = 1,049,827	*n* = 46^c^	
Women's age	15−19	38.2	34.8	In 41% of countries, prevalence is greatest among the 35+ age group.
	20−34	37.8	23.9	
	35+	38.3	41.3	
Residence	Urban	36.3	32.6	In 67% of countries, prevalence is higher in rural areas.
	Rural	38.9	67.4	
Education^d^	None	40.4	53.3	In 46% of counties, prevalence decreases with education.
	Primary	37.4	17.8	
	Secondary	35.7	20.0	
	Higher	32.5	8.9	
Wealth quintile	Lowest	41.0	54.3	In 29% of countries, prevalence decreases by increasing wealth index.
	Second	39.3	15.2	
	Middle	39.1	6.5	
	Fourth	36.9	15.2	
	Highest	34.8	8.7	
Maternity status	Pregnant	44.5	73.9	In 74% of countries, pregnant women have the highest prevalence of anemia.
	BF, not pregnant	39.8	21.7	
	Not BF, not pregnant	36.9	4.3	
Iron supplementation	No	40.4	63.0	In 63% of countries, prevalence is higher among those not having received or bought iron supplementation during pregnancy.
	Yes	38.8	37.0	

a This is a crude weighted average using the overall sample size in each country.

b Unadjusted odds ratios were only run for child sex and child deworming given the descriptive associations to assess significance.

c Angola DHS is not included in the Women's database, hence a lower total number of countries for women.

d Denominator does not include countries with no data reported/collected or for which the sample size was less than 25.

The age categories reported in Table 1 were chosen to ensure adequate sample size and to account for changes in dietary patterns in children, and for adolescent, reproductive, and late reproductive age groupings in women. In children, anemia prevalence decreased with age in 33 of 47 countries. In over 70% of the countries, children 6−11 months old had the highest prevalence of anemia, while in all countries children aged 24−59 months had the lowest prevalence of anemia. In women, there was no strong pattern by age group. In just over 40% of the countries, the prevalence of anemia was highest among those aged 35+; however, in most countries, the percentage difference between the prevalence in the selected age groups was small (less than 5 percentage points).

Anemia prevalence was also disaggregated by gender in children. In over 80% of countries, the prevalence of any anemia was higher among boys. In the majority of countries (35 of 47), there was no significant difference in the odds of anemia for boys compared with girls. However, in 11 of 47 countries, the odds of anemia are greater for boys, whereas no countries showed greater odds for girls. The overall effect showed 5% higher odds of anemia for boys.

In 83% of countries, the prevalence of anemia in children was higher among those living in a rural setting but only in Benin and Azerbaijan, this difference was larger than 5 percentage points. Similarly, in 67% of countries women had a higher prevalence of being anemic when residing in rural areas.

When disaggregated by maternal education, in half of the countries child anemia prevalence decreases by increasing the level of maternal education. With regard to women, anemia follows a similar linear trend. The average prevalence of any anemia among women with no education was 40%, whereas those with the highest level of education had an average prevalence of 33%. In nearly 60% of countries, the prevalence of anemia is highest among those with no formal education.

As with education, the lowest wealth quintile also shows the highest prevalence of being anemic both in children and women, 60% and 41%, respectively. However, a clear linear trend was not observed.

Among pregnant women, the average prevalence of anemia was 45%. Among breastfeeding and nonpregnant, it was 40% and among nonbreastfeeding and nonpregnant women, it was 37%. This indicates that pregnant women are most likely to be anemic in the majority of countries. This is also reflected in the proportion of countries that have highest anemia prevalence in this category (74%).

### Anemia prevalence by other relevant background characteristics

Anemia prevalence was also assessed by key nutrition‐specific interventions, iron supplementation for women and children, and deworming for children. In over 60% of countries, anemia prevalence was highest among those not receiving/buying iron supplements (66% and 63% for children and women, respectively). With regard to deworming, 95% of countries had higher anemia prevalence among children for which no deworming was reported (measured as having taken intestinal parasite drugs in the 6 months preceding the survey). Among this group of children, the average anemia prevalence was nearly 60%. Further, in nearly 60% of countries (27 of 47), the odds of anemia were lower for children reportedly taking deworming medication suggesting a protective effect (no countries showed lower odds for those not deworming).

In addition, we tested age‐ and sex‐adjusted associations (not presented in the tables) between child diarrhea, fever, acute respiratory infection (ARI), and anemia. In 19 of 47 countries (40%), children with reported diarrhea in the last 2 weeks had higher odds of being anemic. Similarly, children in 25 countries (54%) had higher odds of anemia among those with reported fever in the last 2 weeks. Only in seven countries (15%) were children who reported ARI in the last 2 weeks at higher odds of anemia, however in Burkina Faso there appeared to be a reverse trend in the OR. In the multivariate analysis, these variables were not included, for the reasons explained in the discussion below.

### Anemia prevalence by water and sanitation indicators

Any anemia prevalence for children and women was also examined against water and sanitation variables to explore directional trends. On average, across the countries with available data and a sample size above 25, 44% of households used unimproved sanitation facilities and 22% used no sanitation facility (“open”). On the other hand, 21% of households on average used an unimproved water source but over 50% access this from off their premises (30% within 30‐min walking distance, round trip and nearly 20% over 30 min walking, round trip). Annex B (online only) provides additional context, as it illustrates estimates of improved and unimproved water and sanitation indicators by country at the household level.

Table 2 presents averages of any anemia prevalence among children and women by household water and sanitation characteristics. The table also describes the overall directional patterns and associations among the water and sanitation disaggregated categories.

**Table 2:** Weighted averages of any anemia prevalence, and pattern and associations across countries in anemia prevalence among children and women by household water and sanitation characteristics, DHS surveys (2006−2017)

Variable	Categories	Weighted average prevalence of anemia^a^	Percentage of countries with the highest prevalence of anemia by category^b^	Overall anemia prevalence pattern	Associations (see Annex C and D for forest plots, online only)^d^
**Children, 6–59 months of age**		*n* = 385,541	*n* = 47		Adjusted for age and sex
Water access	On‐premises Off‐premises	57.7 51.6	9.1 90.9	In 91% of countries, prevalence is higher for water access off‐premises.	In 27 of 43 countries, the odds of anemia were significantly higher for children accessing water off‐premises.**
Water source	Improved Unimproved Surface	54.3 54.7 61.6	21.7 33.3 53.8	In 54% of countries, prevalence is highest for surface water.	In 12 of 45 and 12 of 40 countries, the odds of anemia were significantly higher for unimproved and surface water, respectively, as compared to improved.**
Sanitation facility	Improved Unimproved Open	52.0 56.7 64.8	6.5 37.8 66.7	In 57% of countries, prevalence decreases from open/no facility to improved facility.	In 18 of 45 and 26 of 40 countries, the odds of anemia were significantly higher for unimproved and open sanitation facilities, respectively.**
Sanitation sharing	Not shared Shared	56.2 52.7	64.4 38.3	In 64% of countries, prevalence is higher for not shared toilet facility.	Only in one of 44 countries were the odds of anemia significantly higher for children using a shared sanitation facility. In six countries using a shared facility was protective.**
**Women, 15–49 years old**		*n* = 1,049,827	*n* = 46^c^		Adjusted for age
Water access	On‐premises Off‐premises	36.5 39.0	27.9 72.1	In 72% of countries, prevalence is higher for water access off‐premises.	In 18 of 43 countries, odds of anemia are significantly higher for access off‐premises.**
Water source	Improved Unimproved Surface	37.6 38.0 39.8	33.3 31.8 42.1	In 42% of countries, the prevalence is highest in the surface water category.	In 10 of 45 and nine of 38 countries, the odds of anemia were significantly higher for unimproved and surface water as compared to improved.**
Sanitation facility	Improved Unimproved Open	36.3 39.0 41.8	13.3 22.7 72.5	In 58% of countries, prevalence decreases from open/no facility to improved facility.	In 14 of 44 and 24 of 40 countries, the odds of anemia were significantly higher for unimproved and open sanitation facilities, respectively.**
Sanitation sharing	Not shared Shared	37.5 36.9	51.1 48.9	In 51% of countries, prevalence is higher among not shared facility users.	In no country, there was a significant increase in odds of anemia for women using a shared toilet, but in four countries using a shared toiled showed a significant protective effect.

a This is a crude weighted average using the overall sample size in each country.

b For some variables, the total percentage across categories does not add to 100% given differences in the denominators across categories (to account for outliers, a country with a variable category with no data or *n* < 25 was removed from the pattern calculations).

c Angola DHS is not included in the women's database, hence a lower total number of countries for women.

d Denominator does not include countries with no data reported/collected or for which the sample size was less than 25.

List of countries included in the denominator can be seen in the forest plots, with ^**^ *P* < 0.05.

#### Water

The prevalence of anemia among children accessing water on‐ and off‐premises was 58% and 52%, respectively, and in 91% of countries, the highest anemia prevalence was indeed among those accessing water off the household premises. Anemia prevalence among children is higher for those living in households using unimproved (55%) or surface water (62%). Further, in the surface water category, 21 of 39 countries showed a higher prevalence of anemia. For women, the average anemia prevalence was 37% and 39% on‐ and off‐premises, respectively, and in 31 of 43 countries, the rate was highest in the off‐premises category.

**Figure 1:**
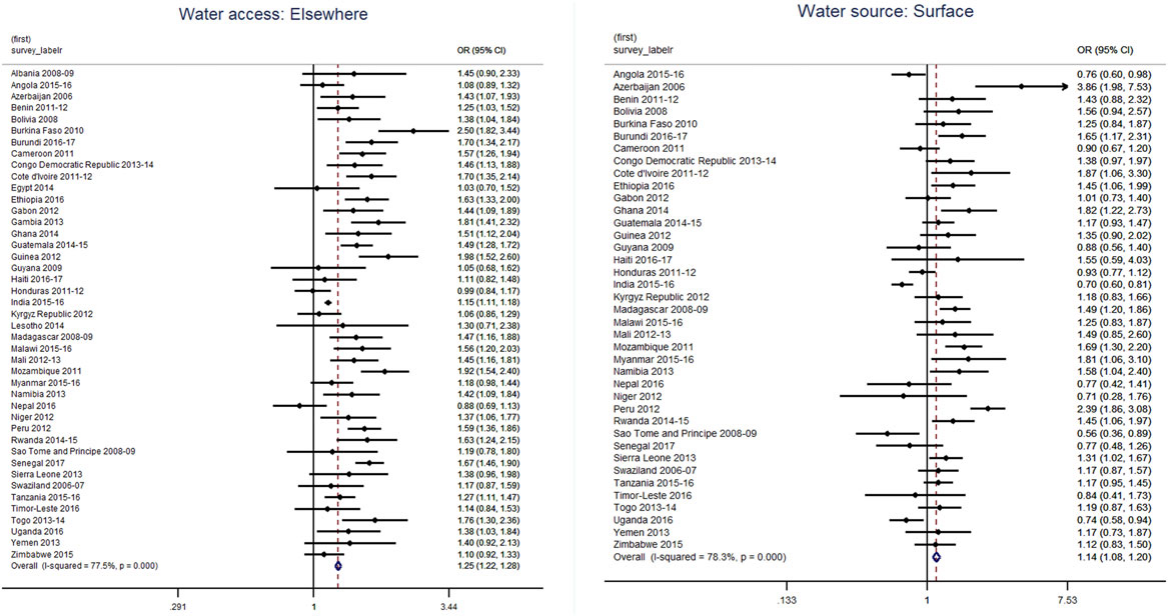
Forest plots illustrating the age‐ and sex‐adjusted odds ratio between anemia and water access and water source among children 6−59 months of age (2006−2017).

Calculation of unadjusted and adjusted ORs found that about a quarter of countries had a statistically significant difference in anemia prevalence, with children exposed to an unimproved water sources having higher odds of being anemic, ranging from 21% higher odds in Tanzania (adjusted OR: 1.21, *P* = 0.03) to two‐fold increase in Mali (adjusted OR: 2.07, *P* < 0.001). Twelve of 40 countries showed higher adjusted odds of anemia among children using surface water, though, in five countries, a protective effect was noted, among which this result was strongest in India (adjusted OR: 0.70, *P* < 0.001; see Figure 1). In both children and women, having access to water on the premises appears to be protective against anemia. A comprehensive global analysis of age‐ and sex‐adjusted ORs for water variables are represented as forest plots and can be found in Annex C (online only) for children and in Annex D (online only) for women.

#### Sanitation

The average prevalence of anemia among children using unimproved or open sanitation facilities was 57% and 65%, respectively. Prevalence of anemia was 53% among children living in a household with a shared sanitation facility as compared with 56% for those using a not‐shared facility. However, only 18 of 47 countries had a higher prevalence of anemia among those using a shared facility. For women, the prevalence of anemia when living in a household with an unimproved or open sanitation facility was 39% and 42%, respectively. The prevalence of anemia among women using shared (37%) and not shared (38%) sanitation facility was about the same.

**Figure 2:**
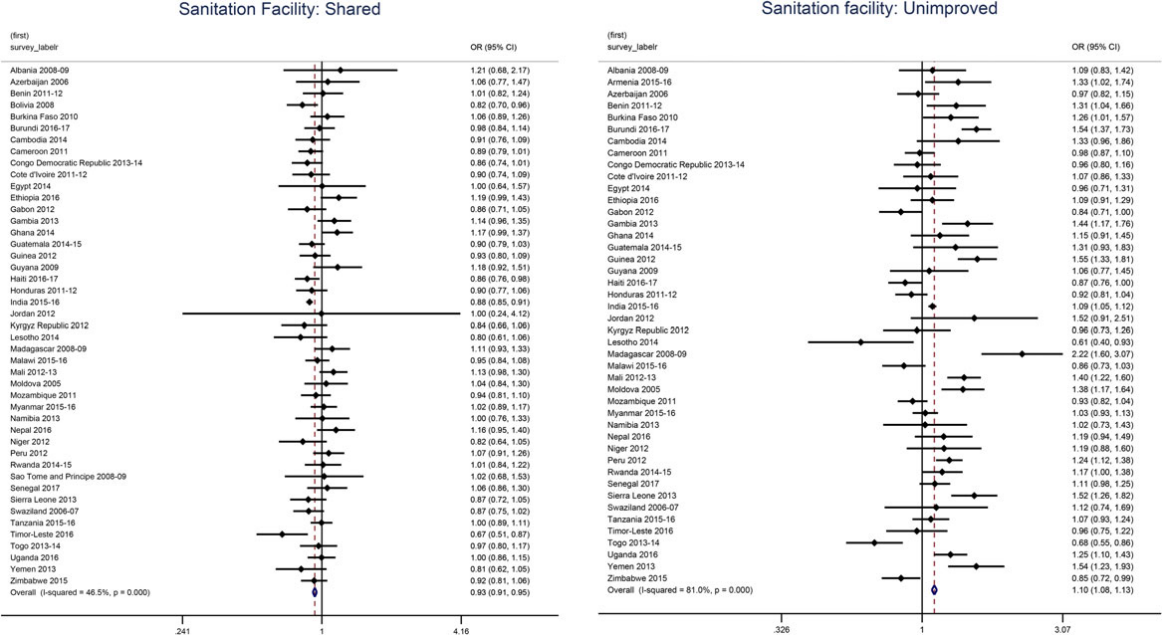
Forest plots illustrating the age‐adjusted odds ratios between anemia and sanitation sharing and sanitation facility among women 15–49 years old (2006−2017).

In 18 of 45 countries, sex‐ and age‐adjusted odds of being anemic among children were higher if they are residing in households with unimproved sanitation facilities. The odds of being anemic for those exposed to unimproved sanitation was highest in Niger (adjusted OR: 2.38, *P* < 0.001) and for children exposed to open or no sanitation facility, in Peru (adjusted OR: 4.44, *P* < 0.001). Among women, Madagascar showed the largest effect of open sanitation on anemia, adjusted OR = 2.63 (*P* < 0.001; see Figure 2).

Across the WASH variables, there appears to be a consistently stronger effect on children as compared with women. In both women and children, the number of countries showing higher adjusted odds of anemia for open defecation is similar (24 of 40 countries and 26 of 40 countries, respectively) suggesting that this is an important factor to look at among both women and children.

Overall data with age‐ and sex‐adjusted ORs can be found in Annex C (online only) for children and in Annex D (online only) for women.

### Multivariate regression results

#### Children

Children living in a household with an unimproved and off‐premises water source showed higher unadjusted odds of being anemic in India (OR: 1.12, *P* < 0.001), Burundi (OR: 1.78, *P* < 0.001), and Senegal (OR: 1.50, *P* < 0.001). However, when adjusting for the relevant background characteristics, including and excluding type of sanitation facility, there is little to no evidence that an unimproved and off‐premises water source is associated with anemia.

Children exposed to surface water did not show a consistent result across countries, and the analysis from India and Burundi showed significant but contrasting effects. From India DHS, there appears to be a protective effect for children exposed to surface water, with 27% lower unadjusted odds of being anemic, whereas for Burundi children exposed to surface water appear to have over two times the odds of being anemic as compared with those unexposed. For India, this association remains protective across the adjusted models that control for relevant factors, however, there appears to be an effect modifier among the covariates, including sanitation as the OR moves further away from the null or no association estimates. For Burundi, the association between anemia and surface water loses significance indicating little to no evidence of a true association.

In all three countries analyzed, the odds of children being anemic when exposed to an unimproved and shared facility increased. For India, this association does not seem to be affected by water source, which is indicated by the limited change in OR between the base model and the water‐adjusted model (1b), and the two fully adjusted models (2b and 3b in Table 3). However, the effect size between the sanitation facility and being anemic reduces from 1.16 (*P* < 0.001) to 1.08 (*P* < 0.05) indicating that the socioeconomic factors, iron supplementation, and deworming may perhaps be confounding the relationship. This is similar in Burundi where the OR drops from 1.54 (*P* < 0.001) to 1.34 (*P* < 0.001) and in Senegal from 1.41 (*P* < 0.001) to 1.16 (*P* < 0.001).

**Table 3:** Multivariate regression results from children data from DHS India 2015−2016, Burundi 2016, and Senegal 2017

		Adjusted OR	*P*‐value	Adjusted OR	*P*‐value	Adjusted OR	*P*‐value
		India 2015−2016		Burundi 2016		Senegal 2017	
Models for “any anemia”	Category	South Asia		East Africa		West Africa	
(0) Base model, unadjusted							
Water source	Unimproved and off‐premises	1.12 (1.08−1.15)	<0.001	1.78 (1.37−2.30)	<0.001	1.50 (1.30−1.72)	<0.001
	Surface	0.73 (0.63−0.85)	<0.001	2.68 (1.78−4.02)	<0.001	0.77 (0.45−1.30)	0.319
Sanitation facility	Unimproved and shared	1.16 (1.10−1.22)	<0.001	1.54 (1.34−1.77)	<0.001	1.41 (1.23−1.62)	<0.001
(1) Minimally adjusted model							
(1a) Water source Adjusted for sanitation facility	Unimproved and off‐premises Surface	0.99 (0.94−1.04) 0.45 (0.36−0.55)	0.618 <0.001	1.74 (1.35−2.24) 2.44 (1.60−3.71)	<0.001 <0.001	1.39 (1.20−1.62) 0.68 (0.31−1.48)	<0.001 0.330
(1b) Sanitation facility Adjusted for water source	Unimproved and shared	1.17 (1.1−1.23)	<0.001	1.45 (1.23−1.71)	<0.001	1.36 (1.17−1.58)	<0.001
(2−3) Fully adjusted models							
(2a) Water source Adjusted for age, sex, residence, education, wealth, iron supplementation, and deworming	Unimproved and off‐premises Surface	1.03 (0.99−1.06) 0.67 (0.57−0.77)	0.135 <0.001	0.80 (0.55−1.16) 1.13 (0.68−1.87)	0.234 0.629	0.98 (0.85−1.14) 0.45 (0.28−0.72)	0.810 0.001
(2b) Sanitation facility Adjusted for age, sex, residence, education, wealth, iron supplementation, and deworming	Unimproved and shared	1.08 (1.02−1.14)	0.008	1.34 (1.15−1.57)	<0.001	1.16 (1.00−1.35)	0.054
(3a) Water source Adjusted for age, sex residence, education, wealth, iron supplementation, deworming, and sanitation facility	Unimproved and off‐premises Surface	0.97 (0.92−1.02) 0.44 (0.36−0.53)	0.220 <0.001	0.834 (0.58−1.21) 1.14 (0.68−1.91)	0.355 0.606	0.98 (0.83−1.15) 0.42 (0.19−0.91)	0.770 0.028
(3b) Sanitation facility Adjusted for age, sex, residence, education, wealth, iron supplementation, deworming, and water source	Unimproved and shared	1.08 (1.02−1.4)	0.006	1.25 (1.04−1.50)	0.019	1.18 (1.00−1.40)	0.049

#### Women

Women living in a household with an unimproved and off‐premises water source have significantly higher odds of being anemic in India (OR: 1.20, *P* < 0.001) and Burundi (OR: 2.31, *P* < 0.001), though no strong evidence can be drawn from Senegal (Table 4). For both India and Burundi, the size of the effect is decreased when including sanitation facilities in the model, bringing the ORs down to 1.15 (*P* < 0.001) and OR: 2.18 (*P* < 0.001). For India, the direction and significance of the OR stays the same also in the fully adjusted model, however, for Burundi, no conclusions can be drawn regarding the association between the unimproved water source and being anemic when adjusting for potential confounders.

**Table 4:** Multivariate regression results from women's data from DHS India 2015−2016, Burundi 2016, and Senegal 2017

		Adjusted OR	*P*‐value	Adjusted OR	*P*‐value	Adjusted OR	*P*‐value
		India 2015−2016		Burundi 2016		Senegal 2017	
Models for “any anemia”	Category	South Asia		East Africa		West Africa	
(0) Base model, unadjusted							
Water source	Unimproved and off‐premises	1.20 (1.18−1.23)	<0.001	2.31 (1.90−2.82)	<0.001	1.10 (9.41−1.28)	0.230
	Surface	1.02 (0.93−1.12)	0.611	2.98 (2.17−4.10)	<0.001	0.47 (0.30−0.74)	0.001
Sanitation facility	Unimproved and shared	1.12 (1.08−1.14)	<0.001	1.38 (1.23−1.55)	<0.001	0.99 (0.84−1.17)	0.919
(1) Minimally adjusted model							
(1a) Water source Adjusted for sanitation facility	Unimproved and off‐premises Surface	1.15 (1.12−1.18) 0.79 (0.71−0.87)	<0.001 <0.001	2.18 (1.79−2.66) 2.61 (1.84−3.69)	<0.001 <0.001	1.05 (0.88−1.26) 0.51 (0.30−0.87)	0.557 0.014
(1b) Sanitation facility Adjusted for water source	Unimproved and shared	1.10 (1.07−1.13)	<0.001	1.41 (1.24−1.60)	<0.001	0.51 (0.30−0.87)	0.014
(2−3) Fully adjusted models							
(2a) Water source Adjusted for age, residence, education, wealth, iron supplementation, and pregnancy status	Unimproved and off‐premises Surface	1.11 (1.07−1.14) 0.95 (0.82−1.09)	<0.001 0.449	0.82 (0.55−1.22) 0.90 (0.53−1.53)	0.328 0.690	0.92 (0.73−1.16) 0.49 (0.23−1.06)	0.477 0.069
(2b) Sanitation facility Adjusted for age, residence, education, wealth, iron supplementation, and deworming	Unimproved and shared	1.03 (0.97−1.08)	0.351	1.18 (1.01−1.38)	0.039	0.87 (0.71−1.07)	0.192
(3a) Water source Adjusted for age, residence, education, wealth, iron supplementation, pregnancy status, and sanitation facility	Unimproved and off‐premises Surface	1.08 (1.03−1.23) 0.77 (0.64−0.91)	0.002 0.003	0.84 (0.56−1.26) 0.87 (0.49−1.53)	0.398 0.621	0.92 (0.72−1.17) 0.43 (0.43−0.93)	0.480 0.032
(3b) Sanitation facility Adjusted for age, residence, education, wealth, iron supplementation, pregnancy status, and water source	Unimproved and shared	1.03 (0.98−1.09)	0.287	1.30 (1.08−1.82)	0.005	0.87 (0.70−1.08)	0.203

Women exposed to surface water, unlike children, in the Indian data set did not show a protective effect for being anemic. However, the unadjusted base model from Burundi and Senegal showed significant but contrasting effects (OR: 2.98, *P* < 0.001; OR: 0.47, *P* < 0.001). For Senegal, the direction and strength of this association remain consistent across fully adjusted models, indicating that it may be a true relationship. For Burundi, there is no strong evidence of an association when adjusting for relevant factors.

When exposure for the unimproved and shared facility is tested, it is significant only for India (OR: 1.12, *P* < 0.001) and Burundi (OR: 1.38, *P* < 0.001) and for both the size of this effect estimate increases when adjusting for the water source. This shows that the variables may be modifying the effect of unimproved and shared facilities. Only for Burundi, does the association remain the same in the fully adjusted model, albeit with some evidence for potential confounding (OR: 1.18, *P* = 0.04).

## Discussion

In 2012, the World Health Assembly (WHA) Resolution 65.6 called for a 50% reduction in anemia among women of reproductive age by 2025 as part of a comprehensive implementation plan on maternal, infant, and young child nutrition.^32^, ^33^ Our analyses of the 47 countries suggest that the global prevalence of anemia remains high (average 55% in children and 38% in women). A review of anemia data in 107 countries by Stevens *et al*. found that between 1995 and 2011, anemia prevalence decreased by 4−5 percentage points in children and pregnant and nonpregnant women.^4^ More updated numbers released in the Global Nutrition Report 2018 suggest that since then, the global prevalence in pregnant women has decreased minimally (41.6% in 2000 to 40.1% in 2016) and has even risen among nonpregnant women (31.1% in 2000 to 32.5% in 2016).^1^ While gains in anemia reduction have been made globally, currently no country is on target to meet the WHA 2025 resolution.^1^ Stevens *et al*. predict that with the current rate of reduction, there is less than a 25% probability, in all regions individually, of meeting the WHA target and the probability at the global level is negligible.^4^

Descriptive sociodemographic results of our study are in line with other studies.^34^, ^35^ In the majority of countries, for children, the highest anemia prevalence was among those 6−11 months of age, those living in rural areas, those having a mother with no education, and those not receiving deworming medication. Additionally, in some countries, boys seem to be more at risk of being anemic compared with girls. In 11 countries, the odds of being anemic was significantly greater for boys compared with girls, with an overall effect across countries suggesting boys have 5% higher odds of being anemic. There were no countries where girls had significantly higher odds of being anemic than boys. A similar pattern has been observed for boys for stunting and wasting prevalence in other studies.^3^, ^36^ To fully understand the reason for the sex difference in child, anemia risk would require deeper investigation; however, some studies have observed similar findings and postulated potential explanations. For instance, a pooled data analysis of four randomized, double‐blind trials for infant zinc and iron supplementation in Southeast Asia similarly found boy infants to be at a higher risk for anemia as well as iron deficiency compared with girl infants.^37^ Further, in infants who did not receive iron supplementation, boys had 60% higher odds of being anemic and over three times the odds of IDA compared with girls by about 11 months of age. While differences were also observed at the baseline (5 months of age at recruitment), they note higher differences in Hb concentration between boys and girls developed during the latter half of infancy. The authors proposed that this is most likely due to a higher iron requirement in boy infants compared with girl infants (particularly in the second half of infancy), which could potentially be explained by a higher growth rate of male infants.^37^ Similar findings were observed in a study of Swedish and Honduran infants with regard to substantial sex differences in Hb concentration and other iron status indicators. The authors suggested several possible explanations for further study, including boys potential lower iron stores at birth, lower iron absorption, greater intestinal loss of iron, and/or being more prone to infections, as compared with girls.^38^

Children in the highest household wealth quintile also had a high anemia prevalence, hence, interventions need to target all children regardless of wealth.^39^, ^40^ Among women, education, residence, and maternity status seem to be the most prominent factors to be considered in anemia reduction program planning.^41^ We suggest increased efforts to reach the most vulnerable groups, to ensure equitable gains in anemia reduction among all subpopulation groups.^42^

There is a growing need to generate evidence regarding the association between WASH and nutrition. A recent WHO, UNICEF, and USAID brief summarizing existing key evidence highlight the potential benefits of integrating WASH interventions into national nutrition policies and programs.^11^ Our study contributes to this body of evidence for understanding the possible association of water and sanitation with anemia. Among the water variables analyzed for their association with anemia, we found that in the majority of countries the highest average prevalence of anemia was among women and children accessing water off‐premises, as well as among those using surface water. In most countries included in the analysis, there was evidence of higher odds of anemia among children who accessed water off‐premises, warranting additional research into the effect of the collection of water off‐premises on anemia, including in situations where the source of off‐premises water is improved.

Across countries, we also found that for both women and children, the average prevalence of anemia did not vastly differ between improved and unimproved water sources. A few other studies that report on water quality and anemia association indicate that children from dwellings with tap water (improved) had, on average, 1.2 g/dL higher Hb than children whose mothers used public wells (unimproved) suggesting a potential effect on anemia.^43^ A study in the Philippines also found water supply to be a significant independent factor affecting Hb levels.^44^

For children, the prevalence of anemia was highest among those using surface water in the majority of countries. However, from the latest India National Family Health Survey 2015−2016, we found instead a protective effect of surface water on being anemic in children. This could possibly be attributed to high iron in surface water, as explored in some studies.^45^ In a study of pregnant and lactating women from Bangladesh, low rates of anemia were attributed to the presence of natural iron in the groundwater.^46^, ^47^

In children, a strong relationship between sanitation facility and anemia is observed, epitomized especially in Madagascar, Burundi, and the Gambia. Counter to what would be expected, the bivariate sanitation tables indicated that not sharing a toilet was associated with the highest anemia prevalence in over half of the countries (for children in 67% and for women in 58% of countries). Furthermore, for children, six countries showed a protective effect of using a shared sanitation facility (while only Niger showed shared sanitation associated with higher odds of anemia). This is in line with a recent meta‐analysis that found that access to community‐level sanitation was associated with better child health outcomes regardless of individual household sanitation facilities.^16^ These studies, in conjunction with our results, imply that children who share a toilet facility are not necessarily at a greater risk of being anemic (and in fact in certain cases may have lower risk). Currently, the JMP classification categorizes shared facilities of any type as “unimproved.” We hypothesize, however, that our observed effect may be related to whether the shared toilet facilities are improved or unimproved, a distinction that should be further explored. Our multivariate regression models for India, Burundi, and Senegal, which combined unimproved and shared facilities as one factor, show that children using unimproved and shared facilities had significantly higher odds of anemia as compared with those using improved and not shared facility. This may reflect that the risk of anemia is influenced more by the unimproved/improved nature of the facility than by whether it is shared. If it is found that sharing or not sharing a toilet facility does not have a strong impact on anemia prevalence compared with if it is an improved facility, this could have programmatic implications for sanitation planning. Another possible explanation may be a limitation of the data in relation to how information is collected on the shared toilet facility. For instance, a household may report a not shared toilet facility but there may be multiple families within that household using the facility.

Among women, the bivariate association between sanitation facilities and anemia was strong and significant in India, Burundi, Sierra Leone, Mali, and Madagascar. A cluster‐randomized efficacy trial investigating effects of improved sanitation facility on BMI and Hb concentration of rural Cambodian pregnant women showed anemia prevalence (Hb < 11.0 g/dL) to be higher among women using nonimproved facilities (34% versus 25%; *P* = 0.04). Even after adjusting for confounders, poor sanitation was associated with lower BMI and Hb concentration among pregnant Cambodian women.^48^

We hypothesize that the direct effects of poor WASH alone may limit nutrient absorption and contribute to anemia.^16^, ^49^, ^50^ The influence of improved WASH on anemia reduction could operate through several possible mechanisms: preventing infections, reducing elevated hepcidin levels, and/or reduced enteropathy causing improved intestinal surface area leading to better iron absorption and reduced loss of nutrients through lower diarrhea prevalence.^12^, ^22^, ^50^ In relation to infection, a recent Cochrane review looking at the interaction between iron and infections concluded that for anemia control, it is necessary to not only focus on iron deficiency but to tackle infections, particularly in settings subject to high‐infection burden.^42^ Our study also shows that in nearly 60% of countries, children who received deworming medication had lower odds of being anemic. Related to environmental enteropathy, a 2014 review by Ngure *et al*. suggests that the minor malabsorption as well as chronic immune stimulation resulting from environmental enteropathy may be a root cause of anemia, particularly anemia of inflammation.^12^ Environmental enteropathy results from infants and young children ingesting large amounts of fecal bacteria, which occurs in areas with poor sanitation and hygiene conditions.^51^

However, three recent trials in Zimbabwe (Sanitation, Hygiene, Infant Nutrition Efficacy project (SHINE)), Kenya, and Bangladesh (WASH Benefits) found that WASH interventions had no effect on linear growth or anemia.^52^, ^53^ Preliminary publications from the WASH Benefits trials reported that nutrition intervention combined with household water, sanitation and handwashing interventions, did not improve child growth more than the nutrition interventions alone.^54^, ^55^ The results from the SHINE trial indicate that there is no added benefit of WASH interventions on anemia when combined with Infant and Young Child Feeding interventions. However, neither the WASH Benefits nor SHINE trials tested improved/on‐plot water supply and sanitation evidence was inconsistent and minimal.^54^, ^55^, ^56^, ^57^ Our analysis provides a useful complement to these trials and insights into further directions for research and programming.

For instance, our analysis found that water access on premises was correlated with lower anemia prevalence and odds, warranting additional research and exploration into the potential causal pathways. Our results also indicate that in some context drinking surface water might be protective due to natural iron in the water. We would strongly suggest investigating this further in India, both at the national and subnational level. Additionally, our exploration of anemia in the context of sanitation further strengthens this body of evidence.

This study is one of the first of its kind to explore the associations between anemia, water source/quality, and sanitation using nationally representative, population‐level, multicountry data.^58^ The strengths of this study include the very large sample sizes analyzed, which increase the accuracy of results. The DHS uses strong quality assurance procedures in the field and during data entry and analysis. Similar quality assurance checks are applied while collecting the biological specimens for anemia testing lending to the accuracy of the data used. Furthermore, this study provides a reference for policymakers and program managers who are in the process of scaling up nutrition with multisectoral planning.

As with any study that uses cross‐sectional surveys, associations may be inferred although causality is not. This study is limited by its dependency on the response of the head of household of the family who typically responds to the water and sanitation questions. The head of household can be a male or a female belonging to one of various age groups identified within the households. Therefore, there could be inconsistencies in the reporting on WASH questions based upon who is responding to the household questionnaire. Further, several associations explored here may be obscuring the presence of a confounding variable. Another potential limitation of this study could be the instrument bias affecting the Hb level assessment. This is likely to be minor since DHS tends to use standardized instruments across surveys, and explicitly shares information where different instruments are used.

In this analysis, despite observing some association, as described in the results, we did not explore the relationship of factors that are strongly affected by temporality (season and year), recall bias, and strong variations between countries. For instance, the association between dietary patterns and nutritional intake with anemia was excluded from the regression analysis, as these data are available for children 6−23 months of age. Similarly, the association between fever, diarrhea, and ARI with anemia was also removed from the pooled analysis and summaries, given the differences in context, year, and season of data collection. Adding such variables to the multivariate regression would potentially obscure the primary relationship of interest between anemia and WASH. It could also introduce issues around the reliability and usability of the results as the DHS collects such data with a 24‐h or 2‐week recall once in 3−5 years.

We recommend expanding this analysis and explore countries with high malaria prevalence, to help identify the most critical factors that independently and strongly affect the differing levels of associations between WASH, anemia, and infection among selected countries. Exploring subnational data for countries could be tedious, but for planning effective and targeted interventions, looking at subnational data would be critical for all the countries. Also, due to time limitation, we could not further explore associations between IDA and WASH variables using the biochemical markers of iron status (e.g., serum ferritin, transferrin saturation, and soluble transferrin receptor) that are available for a couple of DHS surveys. This is another possible expansion of the analysis that could be done as a follow‐up to this study.

## Conclusion

Globally, countries are off‐course in showcasing improvements in anemia prevalence. Many countries still do not have access to basic sanitation facilities and access to water on‐premises. Water and sanitation variables are more strongly associated with anemia prevalence among children compared with women. Not having drinking water access on the premises increases the risk of anemia prevalence in both children and women. A strong relationship between sanitation facility and anemia is also observed in most countries. Such results point toward potential linkages between water and sanitation and anemia. The existing limited evidence highlights that iron interventions alone will not resolve most of the burden of anemia among young children and women. To achieve success in controlling anemia in high infection burden settings, controlling infections by implementing WASH interventions will also be important, in addition to malaria. Hence, implementing nutrition‐sensitive interventions parallel to the nutrition‐specific interventions might hold promise for gains toward anemia reduction.

## Author contributions

M.T.K. conceptualized the study and created the initial analytical design, led interpretation of data, drafting of the manuscript, and led the revision of the intellectual content. A.C. led literature review, contributed to the design, analysis, and interpretation of data, drafting of the manuscript, and supported the intellectual review of the document. A.H. contributed to the design, analysis, and interpretation of the data, drafting of the manuscript, and supporting revision of the intellectual content. T.P. led the acquisition of data, provided inputs to the methodology, and conducted data analysis. D.G. participated in the drafting of the “Methods” section of the manuscript and review of the versions. C.E. reviewed various versions of the document, providing content guidance, and revisiting the intellectual content and approval of the final version of the document.

## Statement

This manuscript was presented at the World Health Organization (WHO) technical consultation “Use and Interpretation of Haemoglobin Concentrations for Assessing Anaemia Status in Individuals and Populations,” held in Geneva, Switzerland on November 29−30 and December 1, 2017. This paper is being published individually but will be consolidated with other manuscripts as a special issue of *Annals of the New York Academy of Sciences*, the coordinators of which were Drs. Maria Nieves Garcia‐Casal and Sant‐Rayn Pasricha. The special issue is the responsibility of the editorial staff of *Annals of the New York Academy of Sciences*, who delegated to the coordinators preliminary supervision of both technical conformity to the publishing requirements of *Annals of the New York Academy of Sciences* and general oversight of the scientific merit of each article. The workshop was supported by WHO, the Centers for Disease Control and Prevention (CDC), the United States Agency for International Development (USAID), and the Bill & Melinda Gates Foundation. The authors alone are responsible for the views expressed in this paper; they do not necessarily represent the views, decisions, or policies of the WHO. The opinions expressed in this publication are those of the authors and are not attributable to the sponsors, publisher, or editorial staff of *Annals of the New York Academy of Sciences*.

## Competing interests

The authors declare no competing interests.

## Supporting information


**Annex A**. Prevalence estimates of anemia among children 6–59 months of age and women 15–49 years old for countries with standardized anemia data, DHS 2006 and 2018.
**Annex B**. Percentage distribution of water and sanitation indicators, DHS surveys (2006–2018).

**Annex C**. Forest plots illustrating the adjusted odds ratios for anemia in children 6–59 months of age, for categories of water and sanitation indicators.
**Annex D**. Forest plots illustrating the adjusted odds ratios for anemia in women 15–49 years old, for categories of WASH indicators.

## Footnotes

1 Nutrition‐specific interventions address the immediate determinants of malnutrition. The 2013 Lancet Series on Maternal and Child Nutrition recommends 10 direct interventions, such as micronutrient supplementation or fortification, acute malnutrition treatment and exclusive breastfeeding, and complementary feeding of young children.

2 Nutrition‐sensitive interventions address the underlying causes of undernutrition. They include actions from a range of sectors, including health, agriculture and food systems, WASH promotion, education and social protection. Examples of such interventions include, improving the purchasing power of women, improving access to food, diversifying agriculture, advancing biofortification, promoting healthy diets, supporting breastfeeding, and improving access to WASH.
