# Supplementary material for: Exploring associations between water, sanitation, and anemia through 47 nationally representative demographic and health surveys
Source: Ann N Y Acad Sci. 2019 Jun 24;1450(1):249–67. doi: 10.1111/nyas.14109 (PMC6771505; doi:10.1111/nyas.14109)
Supplement: Supplementary file 1 — Annex A. Prevalence estimates of anemia among children 6–59 months of age and women 15–49 years old for countries with standardized anemia data, DHS 2006 and 2018. Annex B. Percentage distribution of water and sanitation indicators, DHS surveys (2006–2018). [file NYAS-1450-249-s001.docx]

**Supplemental Tables**

**Annex A. Prevalence estimates of anemia among children 6-59 months of age and women 15 to 49 years old for countries with standardized anemia data, DHS 2006 and 2018**

|  |  | **Children** | | | | | **Women^[[1]](#footnote-1)^** | | | | |
| --- | --- | --- | --- | --- | --- | --- | --- | --- | --- | --- | --- |
| **Country** | **Year** | **N** | **Any**  **anemia (<11.0 g/dl)** | **Mild**  **Anemia**  **(10.0-11.9 g/dl)** | **Moderate Anemia**  **(7.0 - 9.9 g/dl)** | **Severe Anemia**  **(<7.0 g/dl)** | **N** | **Any**  **anemia (<12.0 g/dl)** | **Mild**  **Anemia**  **(10.0-11.9 g/dl)** | **Moderate Anemia**  **(7.0 - 9.9 g/dl)** | **Severe Anemia**  **(<7.0 g/dl)** |
| Albania | 2008 | 1304 | 17.6 | 11.3 | 6.2 | 0.1 | 7443 | 19.0 | 16.3 | 2.6 | 0.1 |
| Angola | 2015 | 5221 | 65.3 | 31.0 | 32.0 | 2.2 | n.a. | n.a. | n.a. | n.a. | n.a. |
| Armenia | 2015 | 1,294 | 15.8 | 11.1 | 4.3 | 0.4 | 5769 | 13.4 | 12.0 | 1.3 | 0.2 |
| Azerbaijan | 2006 | 1818 | 39.3 | 21.4 | 17.4 | 0.5 | 8124 | 37.0 | 29.0 | 7.2 | 0.8 |
| Benin | 2011 | 3267 | 58.8 | 26.2 | 29.5 | 3.1 | 5038 | 41.4 | 32.4 | 8.5 | 0.5 |
| Bolivia (Plurinational State of) | 2008 | 2337 | 62.0 | 25.1 | 33.5 | 3.4 | 5704 | 38.3 | 29.6 | 8.2 | 0.5 |
| Burkina Faso | 2010 | 6043 | 87.9 | 18.1 | 58.5 | 11.3 | 8424 | 48.8 | 33.8 | 14.0 | 1.1 |
| Burundi | 2016 | 5588 | 60.9 | 24.6 | 32.7 | 3.6 | 8587 | 39.3 | 29.0 | 9.6 | 0.6 |
| Cambodia | 2014 | 3955 | 56.6 | 29.7 | 26.4 | 0.5 | 11286 | 45.4 | 38.4 | 6.7 | 0.2 |
| Cameroon | 2011 | 4589 | 61.3 | 28.1 | 31.4 | 1.7 | 7803 | 39.5 | 30.2 | 8.7 | 0.6 |
| Congo, Democratic Republic | 2013 | 7164 | 60.1 | 25.2 | 31.8 | 3.1 | 5364 | 54.2 | 29.6 | 8.4 | 0.3 |
| Côte d'Ivoire | 2011 | 2693 | 75.7 | 25.2 | 47.0 | 3.5 | 4589 | 53.7 | 38.9 | 14.3 | 0.5 |
| Egypt | 2014 | 4533 | 27.4 | 17.8 | 9.6 | 0.0 | 7161 | 25.2 | 23.1 | 2.1 | 0.0 |
| Ethiopia | 2016 | 8482 | 57.6 | 25.1 | 29.4 | 3.1 | 14923 | 23.6 | 17.8 | 5.0 | 0.8 |
| Gabon | 2012 | 2513 | 61.3 | 30.3 | 29.0 | 1.9 | 5267 | 60.6 | 41.3 | 18.4 | 0.9 |
| Gambia | 2013 | 2829 | 71.5 | 24.8 | 42.7 | 4.1 | 4489 | 59.0 | 40.5 | 16.7 | 1.8 |
| Ghana | 2014 | 2312 | 66.8 | 27.0 | 37.4 | 2.4 | 4644 | 42.4 | 32.2 | 9.8 | 0.4 |
| Guatemala | 2014 | 10477 | 32.8 | 20.4 | 12.1 | 0.2 | 25484 | 13.6 | 11.4 | 2.1 | 0.2 |
| Guinea | 2012 | 2802 | 77.6 | 23.6 | 46.1 | 7.9 | 4726 | 49.1 | 35.5 | 12.7 | 0.9 |
| Guyana | 2009 | 1200 | 39.3 | 23.5 | 15.4 | 0.4 | 4595 | 37.4 | 28.6 | 8.3 | 0.6 |
| Haiti | 2016 | 4708 | 66.9 | 28.5 | 36.3 | 2.1 | 9486 | 49.0 | 35.6 | 12.0 | 1.4 |
| Honduras | 2011 | 7894 | 29.3 | 19.3 | 9.8 | 0.3 | 21440 | 15.1 | 13.1 | 1.9 | 0.1 |
| India | 2015 | 200530 | 58.7 | 27.8 | 29.3 | 1.6 | 679445 | 53.1 | 39.6 | 12.4 | 1.0 |
| Jordan | 2012 | 5179 | 32.2 | 19.9 | 12.1 | 0.2 | 6823 | 37.3 | 28.6 | 8.3 | 0.4 |
| Kyrgyzstan | 2012 | 3364 | 42.9 | 22.5 | 19.0 | 1.4 | 8001 | 35.2 | 26.0 | 8.4 | 0.8 |
| Lesotho | 2014 | 1138 | 54.1 | 25.1 | 27.1 | 1.8 | 3297 | 27.3 | 20.1 | 6.5 | 0.6 |
| Madagascar | 2008 | 4845 | 50.2 | 29.7 | 19.6 | 0.8 | 8308 | 35.3 | 29.2 | 5.7 | 0.4 |
| Malawi | 2015 | 4677 | 63.0 | 26.2 | 34.9 | 2.0 | 7933 | 32.7 | 25.1 | 7.0 | 0.6 |
| Mali | 2012 | 4217 | 81.9 | 20.8 | 51.8 | 9.3 | 5172 | 51.4 | 36.8 | 13.3 | 1.3 |
| Mozambique | 2011 | 4640 | 69.2 | 26.1 | 39.1 | 4.0 | 13571 | 54.0 | 38.6 | 14.0 | 1.4 |
| Myanmar | 2015 | 3031 | 59.2 | 31.7 | 26.8 | 0.6 | 12489 | 46.5 | 37.6 | 8.4 | 0.5 |
| Namibia | 2013 | 1412 | 49.6 | 24.6 | 24.2 | 0.8 | 4242 | 20.7 | 16.6 | 3.6 | 0.5 |
| Nepal | 2016 | 2107 | 52.2 | 25.9 | 25.9 | 0.5 | 6414 | 40.8 | 33.5 | 7.0 | 0.3 |
| Niger | 2012 | 4549 | 73.7 | 27.3 | 43.7 | 2.7 | 5060 | 45.8 | 32.7 | 12.2 | 0.9 |
| Peru | 2012 | 7567 | 32.7 | 20.6 | 11.8 | 0.3 | 23373 | 17.7 | 15.1 | 2.4 | 0.1 |
| Republic of Moldova | 2005 | 1311 | 30.7 | 20.5 | 10.2 | 0.0 | 7138 | 27.9 | 23.3 | 4.3 | 0.3 |
| Rwanda | 2014 | 3283 | 36.6 | 20.6 | 15.3 | 0.7 | 6680 | 19.2 | 15.7 | 3.4 | 0.2 |
| Sao Tome and Principe | 2008 | 1409 | 63.6 | 32.7 | 29.7 | 1.3 | 2502 | 43.1 | 34.0 | 8.5 | 0.5 |
| Senegal | 2017 | 8917 | 71.8 | 28.9 | 40.1 | 2.8 | 7906 | 54.1 | 40.4 | 12.7 | 1.0 |
| Sierra Leone | 2013 | 4168 | 80.0 | 26.7 | 47.2 | 6.1 | 7869 | 44.8 | 34.5 | 9.7 | 0.6 |
| Swaziland | 2006 | 1813 | 44.4 | 22.9 | 20.7 | 0.9 | 4598 | 30.4 | 22.5 | 7.6 | 0.3 |
| Timor-Leste | 2016 | 1802 | 41.7 | 28.6 | 12.7 | 0.3 | 4201 | 22.7 | 18.5 | 3.7 | 0.5 |
| Togo | 2013 | 2771 | 70.9 | 25.6 | 42.7 | 2.6 | 4782 | 48.1 | 36.1 | 11.0 | 1.0 |
| Uganda | 2016 | 3893 | 53.8 | 24.4 | 27.2 | 2.2 | 5988 | 31.7 | 25.1 | 6.0 | 0.6 |
| United Republic of Tanzania | 2015 | 7828 | 58.7 | 26.7 | 30.3 | 1.7 | 13064 | 44.8 | 32.8 | 11.1 | 0.9 |
| Yemen | 2013 | 3715 | 86.6 | 14.2 | 56.8 | 15.5 | 7401 | 70.7 | 38.0 | 29.4 | 3.2 |
| Zimbabwe | 2015 | 4354 | 38.1 | 22.3 | 15.3 | 0.5 | 9235 | 26.8 | 20.1 | 6.1 | 0.5 |

Annex B. Percentage distribution of water and sanitation indicators, DHS surveys (2006-2018)

|  |  | **Household Sanitationⁱ** | | **Household water source and access** | | | | |  |
| --- | --- | --- | --- | --- | --- | --- | --- | --- | --- |
| **Country** | **Year** | % of households with an **improved** toilet facility | % of households with an **unimproved** toilet facility | % of households using an **improved** water source | % of households using an **unimproved** water source | % of households with water o**n the premises** | **% of households with water less than 30 minutes awayⁱⁱ** | % of households with water **30 minutes or longer** **away** | Number of households |
| Albania | 2008 | 94.3 | 5.7 | 94.5 | 4.9 | 86.4 | 9.3 | 4.1 | 7999 |
| Angola | 2015 | 47.1 | 52.9 | 52.3 | 45.9 | 34.5 | 33.4 | 28.3 | 16109 |
| Armenia | 2015 | 77.8 | 22.1 | 98.4 | 1.6 | 97.6 | 1.5 | 0.2 | 7893 |
| Azerbaijan | 2006 | 84.7 | 15.2 | 80.3 | 18.5 | 75.9 | 14.7 | 8.4 | 7180 |
| Benin | 2011 | 33.5 | 66.4 | 78.4 | 21.5 | 38.6 | 42.9 | 14.1 | 17422 |
| Bolivia (Plurinational State of) | 2008 | N.A. | N.A. | 85.6 | 13.1 | 88.0 | 9.7 | 2.2 | 19564 |
| Burkina Faso | 2010 | 48.0 | 52.0 | 76.6 | 23.3 | 16.6 | 44.9 | 33.8 | 6448 |
| Burundi | 2016 | 49.8 | 50.2 | 82.9 | 17.0 | 9.6 | 48.2 | 42.2 | 15977 |
| Cambodia | 2014 | 55.0 | 45.0 | 65.2 | 34.8 | N.A. | N.A. | N.A. | 15825 |
| Cameroon | 2011 | 59.1 | 40.9 | 70.8 | 28.4 | 18.8 | 53.6 | 26.5 | 14214 |
| Congo, Democratic Republic | 2013 | 39.6 | 60.4 | 48.7 | 51.0 | 7.1 | 41.3 | 50.8 | 18171 |
| Côte d'Ivoire | 2011 | 46.5 | 53.4 | 78.3 | 20.9 | 44.2 | 38.6 | 16.6 | 9686 |
| Egypt | 2014 | 77.8 | 22.1 | 97.8 | 1.8 | 92.7 | 5.3 | 1.9 | 28175 |
| Ethiopia | 2016 | 14.8 | 85.2 | 64.8 | 35.1 | 20.1 | 35.3 | 44.5 | 16650 |
| Gabon | 2012 | 64.3 | 35.7 | 93.0 | 6.2 | 65.6 | 17.3 | 15.6 | 9755 |
| Gambia | 2013 | 60.8 | 39.1 | 91.0 | 8.1 | 46.1 | 42.3 | 10.9 | 6217 |
| Ghana | 2014 | 74.2 | 25.8 | 86.3 | 13.7 | 39.6 | 49.6 | 9.2 | 5841 |
| Guatemala | 2014 | 91.4 | 8.6 | 57.3 | 41.8 | 89.5 | 8.2 | 2.3 | 21383 |
| Guinea | 2012 | 44.2 | 55.8 | 74.9 | 25.1 | 22.9 | 43.6 | 32.8 | 7109 |
| Guyana | 2009 | 93.0 | 7.0 | 94.6 | 4.7 | 92.6 | 5.7 | 1.2 | 5632 |
| Haiti | 2016 | 55.3 | 44.8 | 74.0 | 26.0 | 14.0 | 55.8 | 30.1 | 13405 |
| Honduras | 2011 | 77.1 | 22.9 | 89.8 | 9.2 | 88.4 | 9.6 | 1.9 | 21362 |
| India | 2015 | 57.5 | 42.4 | 89.9 | 9.9 | 66.3 | 27.2 | 6.4 | 601509 |
| Jordan | 2012 | 99.9 | 0.0 | 98.9 | 1.1 | N.A. | N.A. | N.A. | 15190 |
| Kyrgyzstan | 2012 | 98.5 | 1.5 | 88.2 | 11.6 | 68.2 | 27.7 | 3.4 | 8040 |
| Lesotho | 2014 | 71.6 | 28.4 | 83.6 | 16.4 | 26.9 | 45.6 | 26.4 | 9402 |
| Madagascar | 2008 | 15.0 | 85.0 | 43.8 | 56.0 | 27.3 | 52.9 | 17.3 | 11284 |
| Malawi | 2015 | 83.0 | 17.1 | 87.2 | 12.7 | 15.4 | 40.7 | 42.9 | 26361 |
| Mali | 2012 | 45.5 | 54.5 | 70.0 | 29.4 | 27.5 | 61.6 | 9.2 | 4240 |
| Mozambique | 2011 | 20.5 | 79.6 | 51.0 | 48.8 | 15.2 | 43.0 | 39.4 | 13919 |
| Myanmar | 2015 | 58.1 | 41.9 | 80.2 | 19.6 | 36.8 | 56.1 | 6.2 | 12500 |
| Namibia | 2013 | 48.9 | 51.1 | 86.9 | 10.4 | 53.3 | 30.7 | 14.8 | 9849 |
| Nepal | 2016 | 83.7 | 16.3 | 94.6 | 5.4 | 68.9 | 26.0 | 5.1 | 11040 |
| Niger | 2012 | 18.7 | 81.3 | 67.0 | 32.9 | 10.7 | 39.7 | 47.8 | 10750 |
| Peru | 2012 | 8.1 | 91.9 | 87.7 | 7.7 | 78.4 | 17.7 | 3.8 | 27218 |
| Republic of Moldova | 2005 | 82.7 | 17.3 | 92.1 | 7.9 | N.A. | N.A. | N.A. | 11095 |
| Rwanda | 2014 | 71.2 | 28.8 | 72.9 | 27.0 | 10.5 | 40.7 | 48.7 | 12699 |
| Sao Tome and Principe | 2008 | 37.6 | 62.4 | 94.2 | 5.8 | 25.5 | 46.3 | 24.5 | 3536 |
| Senegal | 2017 | 72.7 | 27.4 | 81.0 | 18.9 | 69.9 | 23.0 | 6.6 | 8380 |
| Sierra Leone | 2013 | 49.0 | 51.0 | 60.6 | 39.1 | 9.4 | 62.4 | 26.4 | 12629 |
| Swaziland | 2006 | 16.2 | 83.8 | 69.8 | 29.9 | 43.0 | 32.0 | 24.5 | 4843 |
| Timor-Leste | 2016 | 58.6 | 41.4 | 78.6 | 21.4 | 65.9 | 17.5 | 14.2 | 11502 |
| Togo | 2013 | 43.3 | 56.7 | 64.1 | 35.8 | 16.0 | 59.4 | 24.1 | 9549 |
| Uganda | 2016 | 38.8 | 61.2 | 78.3 | 21.5 | 18.7 | 34.0 | 46.8 | 19588 |
| United Republic of Tanzania | 2015 | 35.5 | 64.5 | 61.4 | 38.5 | 28.6 | 31.0 | 40.4 | 12563 |
| Yemen | 2013 | 48.9 | 51.2 | 58.8 | 39.8 | 87.8 | 1.0 | 10.4 | 17351 |
| Zimbabwe | 2015 | 66.8 | 33.2 | 78.1 | 21.8 | 37.6 | 33.3 | 28.6 | 10534 |
| ⁱThis data has been extracted from the STAT compiler and as such disaggregation by shared toilet was not possible. Improved sanitation combines shared and not shared, and unimproved is all shared. For further analysis in this paper, we have two separate variables (1) for sanitation type - improved, unimproved, none; and (2) shared sanitation - yes, no.  ⁱⁱFor the analysis conducted in this paper, household water source outside of the compound was grouped as, “water source away from premises” to avoid small sample sizes | | | | | | | | | |

1. For pregnant women the following anemia classification is used: Any <11.0 g/dl; Mild 10.0-10.9 g/dl; Moderate 7.0-9.9 g/dl; Severe < 7.0 g/dl [↑](#footnote-ref-1)
