# Supplementary material for: Exploring associations between water, sanitation, and anemia through 47 nationally representative demographic and health surveys
Source: Ann N Y Acad Sci. 2019 Jun 24;1450(1):249–67. doi: 10.1111/nyas.14109 (PMC6771505; doi:10.1111/nyas.14109)
Supplement: Supplementary file 2 — Annex C. Forest plots illustrating the adjusted odds ratios for anemia in children 6–59 months of age, for categories of water and sanitation indicators. Annex D. Forest plots illustrating the adjusted odds ratios for anemia in women 15–49 years old, for categories of WASH indicators. [file NYAS-1450-249-s002.docx]

Supplemental Figures

## Annex C – Forest plots illustrating the adjusted odds ratios for anemia in children 6 to 59 months of age, for categories of water and sanitation indicators

Forest Plot 1. Association between anemia and water access on premise and off premise (Elsewhere) in children 6 to 59 months old, DHS (2006-2017)
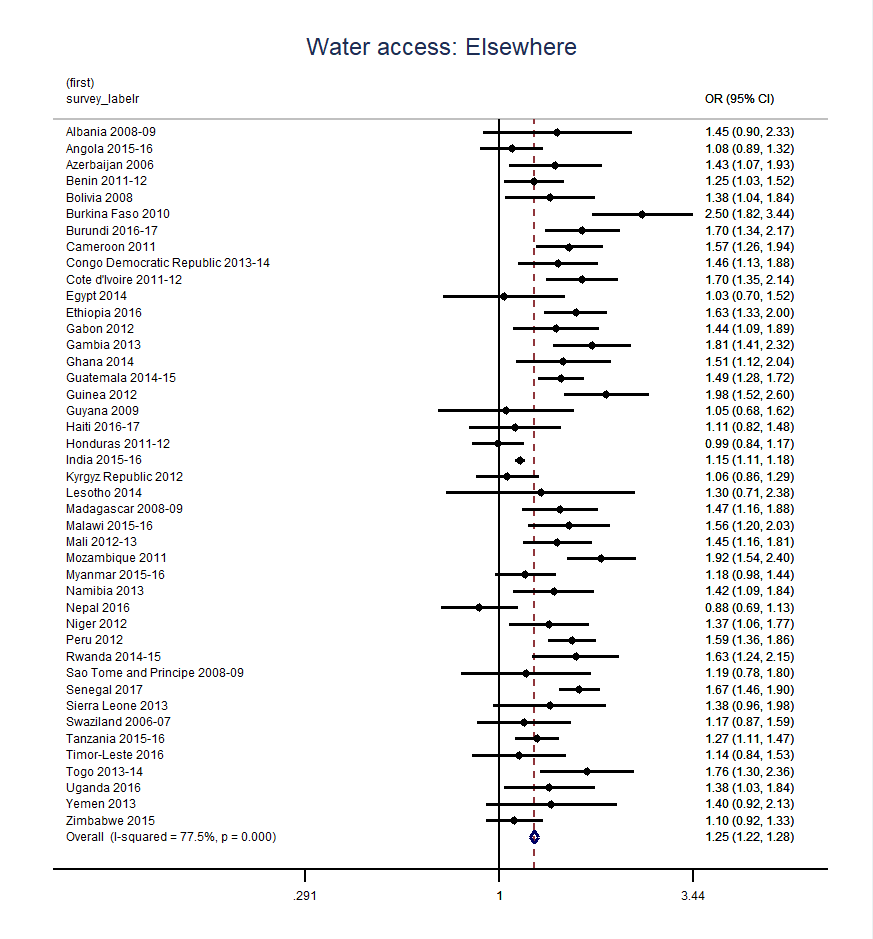


**Country**

The cumulative age and sex adjusted OR for anemia in children exposed to off-premise water was 1.25 (1.22-2.28), with an overall level of heterogeneity of 77.5%, p<0.001

Forest Plot 2. Association between anemia and water source improved and unimproved in children 6 to 59 months old, DHS (2006-2017)
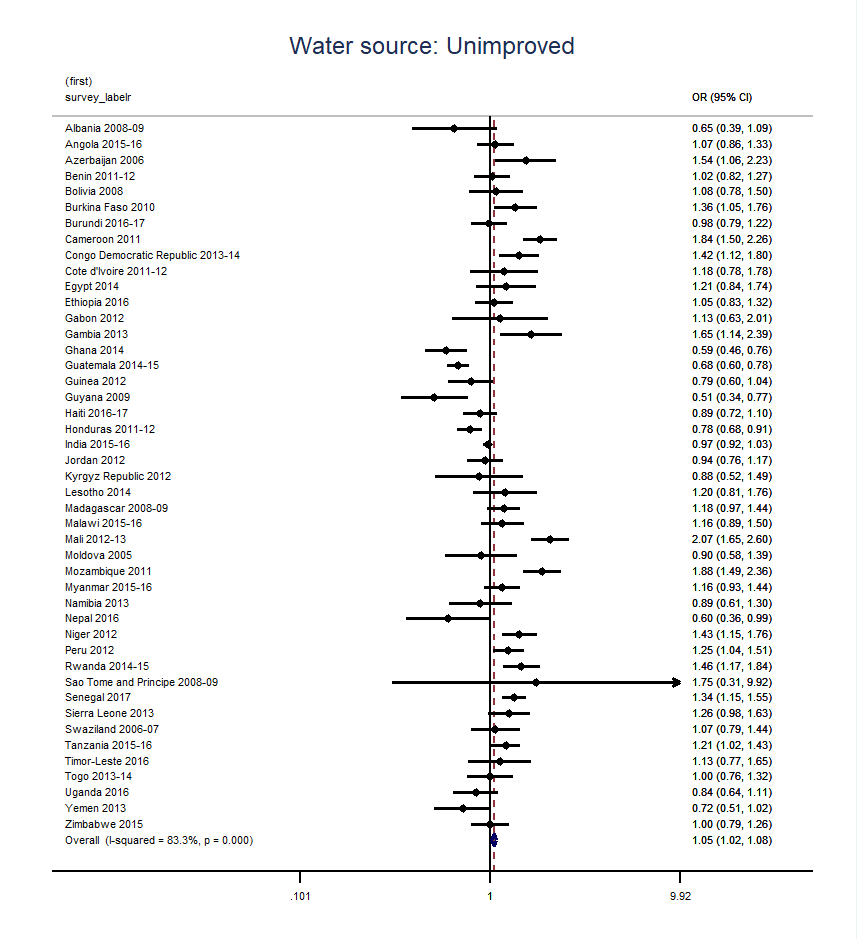


**Country**

The cumulative age and sex adjusted OR for anemia in children exposed to unimproved water source was 1.05 (1.02-1.08), with an overall level of heterogeneity of 83.3%, p<0.001

Forest Plot 3. Association between anemia and water source improved and surface water in children 6 to 59 months old, DHS (2006-2017)
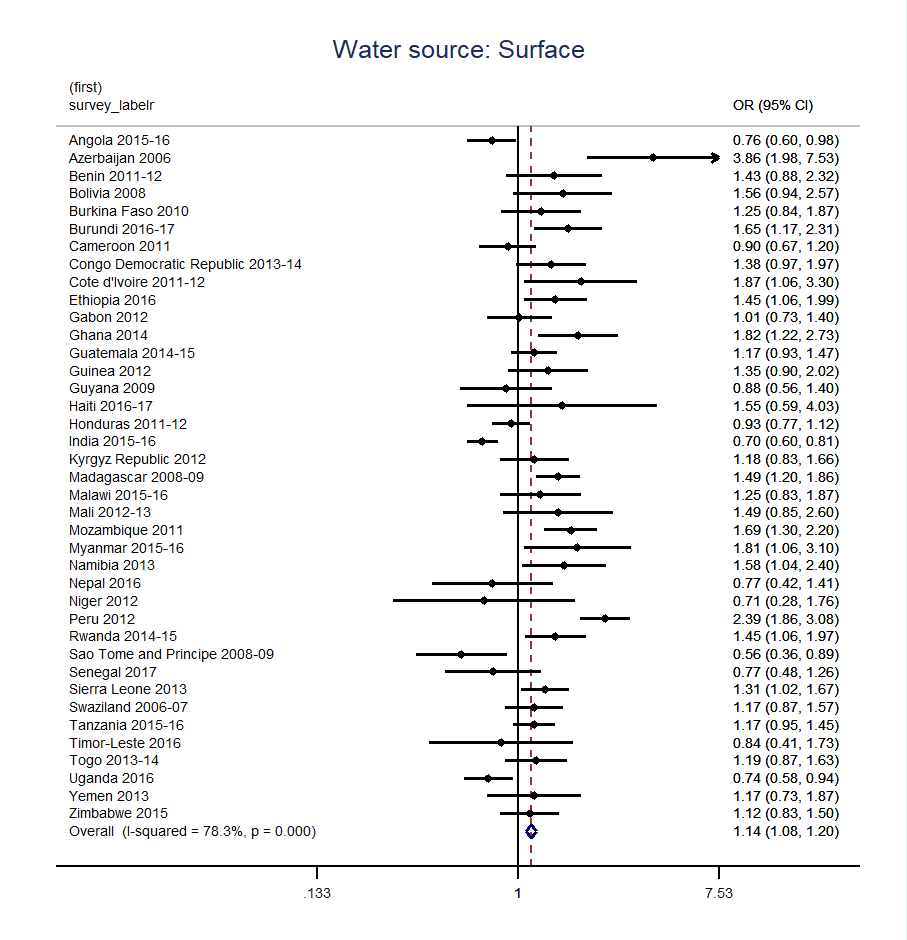


**Country**

The cumulative age and sex adjusted OR for anemia in children exposed to surface water was 1.14 (1.08-1.20), with an overall level of heterogeneity of 78.3%, p<0.001

Forest Plot 4. Association between anemia and private and shared sanitation facility in children 6 to 59 months old, DHS (2006-2017)
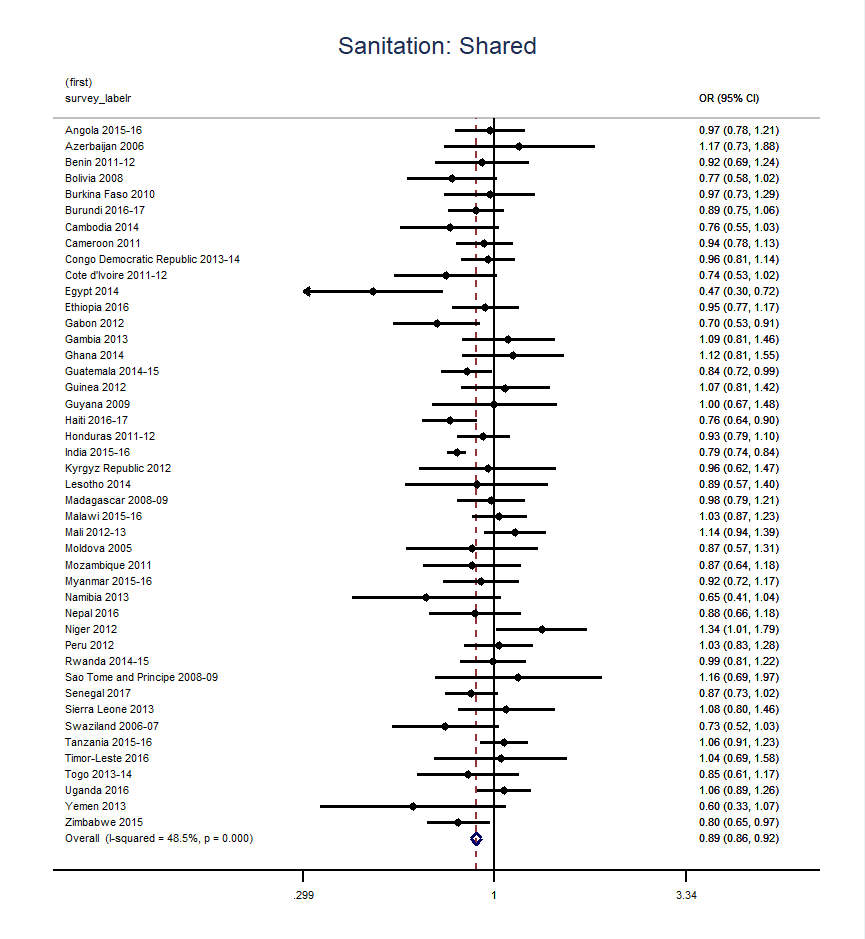


**Country**

The cumulative age and sex adjusted OR for anemia in children exposed to a shared sanitation facility was 0.89 (0.86-0.92), with an overall level of heterogeneity of 48.5%, p<0.001

Forest Plot 5. Association between anemia improved and unimproved sanitation facility in children 6 to 59 months old, DHS (2006-2017)


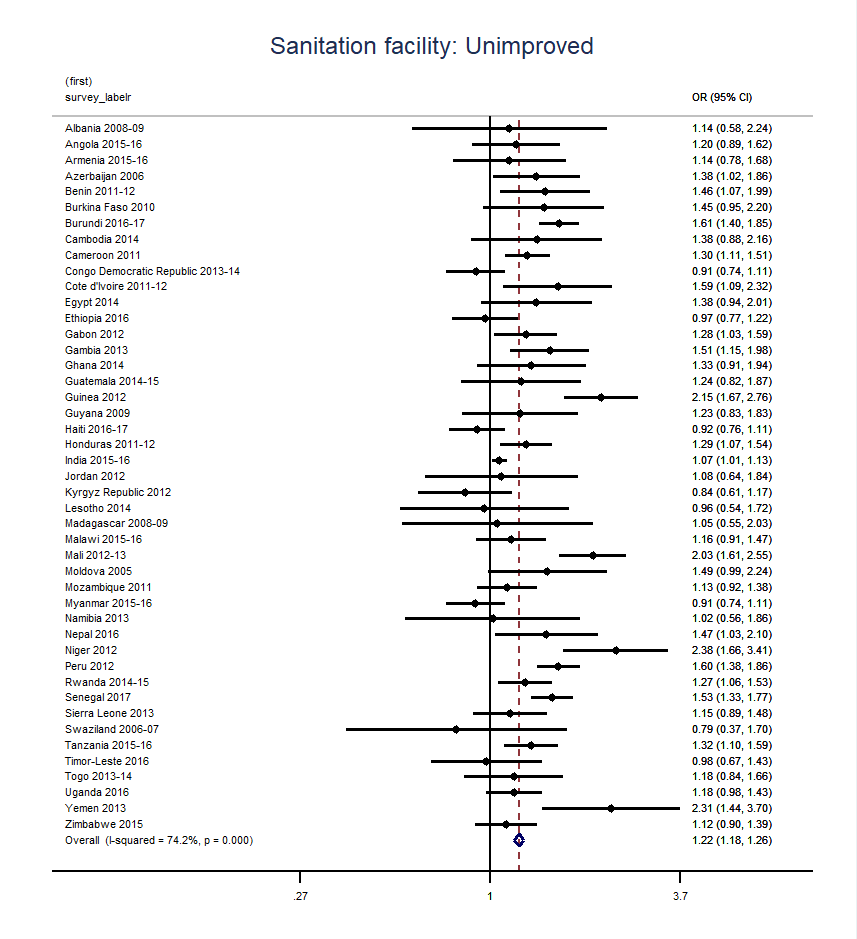


**Country**

The cumulative age and sex adjusted OR for anemia in children exposed to a unimproved sanitation facility was 1.22 (1.18-1.26), with an overall level of heterogeneity of 74.2%, p<0.001

Forest Plot 6. Association between anemia improved and open sanitation facility in children 6 to 59 months old, DHS (2006-2017)


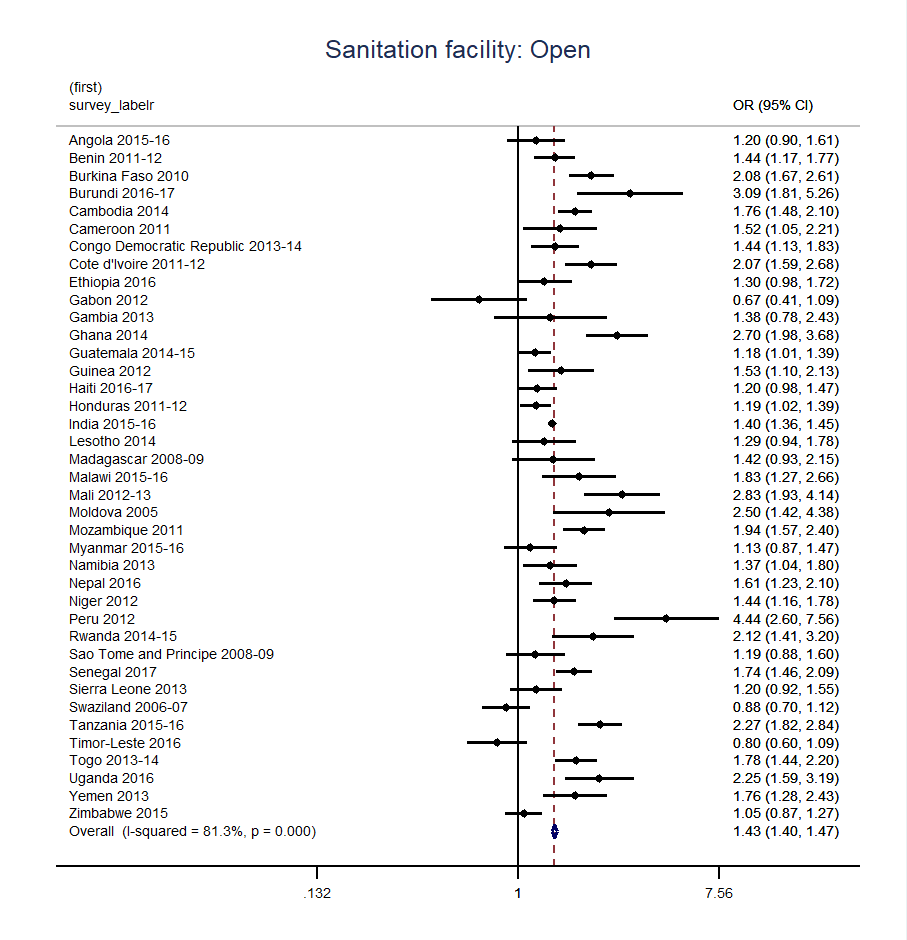


**Country**

The cumulative age and sex adjusted OR for anemia in children exposed to an open sanitation facility was 1.43 (1.40-1.47), with an overall level of heterogeneity of 81.3%, p<0.001

## Annex D– Forest plots illustrating the adjusted odds ratios for anemia in women 15 to 49 years old, for categories of WASH indicators

Forest Plot 1. Association between anemia and water access on premise and off premise (Elsewhere) in women 15 to 49 years old, DHS (2006-2017)
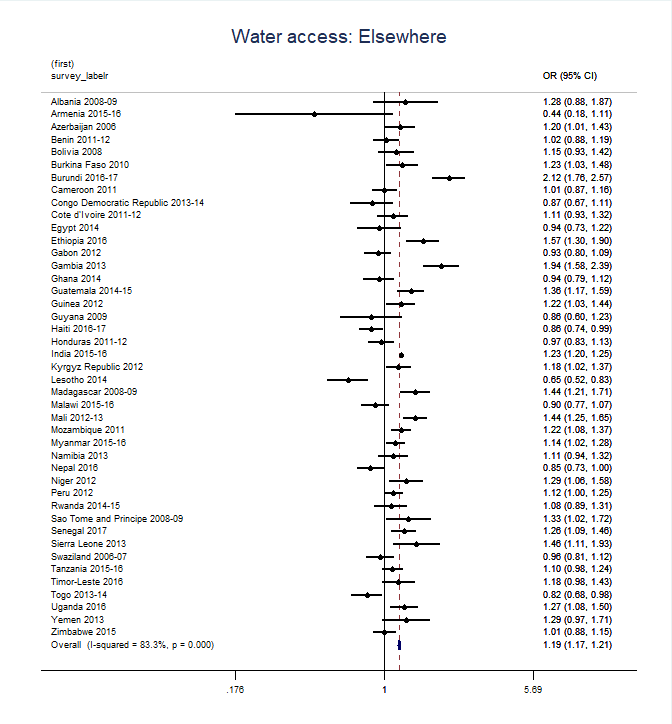


**Country**

The cumulative age adjusted OR for anemia in women exposed to off-premise water was 1.19 (1.17-1.21), with an overall level of heterogeneity of 83.3%, p<0.001

Forest Plot 2. Association between anemia and water source improved and unimproved in women 15 to 49 years old, DHS (2006-2017)


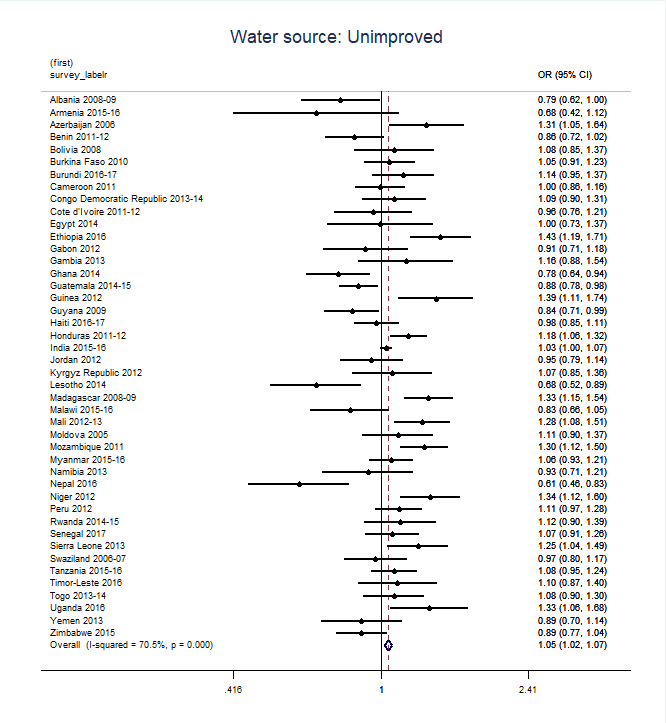


**Country**

The cumulative age adjusted OR for anemia in women exposed to unimproved water source was 1.05 (1.02-1.07), with an overall level of heterogeneity of 70.5%, p<0.001

Forest Plot 3. Association between anemia and water source improved and surface water in women 15 to 49 years old, DHS (2006-2017)
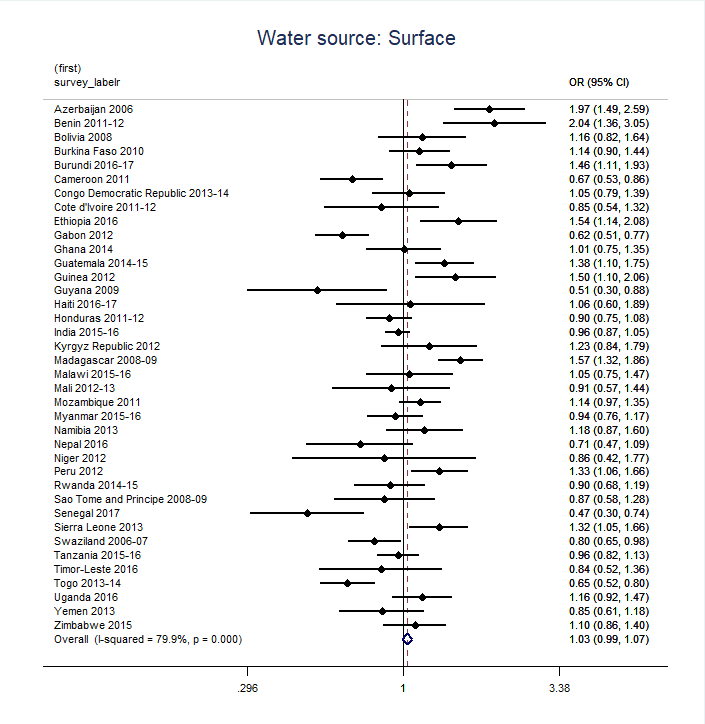


**Country**

The cumulative age adjusted OR for anemia in women exposed to surface water was 1.03 (0.99-1.07), with an overall level of heterogeneity of 79.9%, p<0.001

Forest Plot 4. Association between anemia and private and shared sanitation facility in women 15 to 49 years old, DHS (2006-2017)
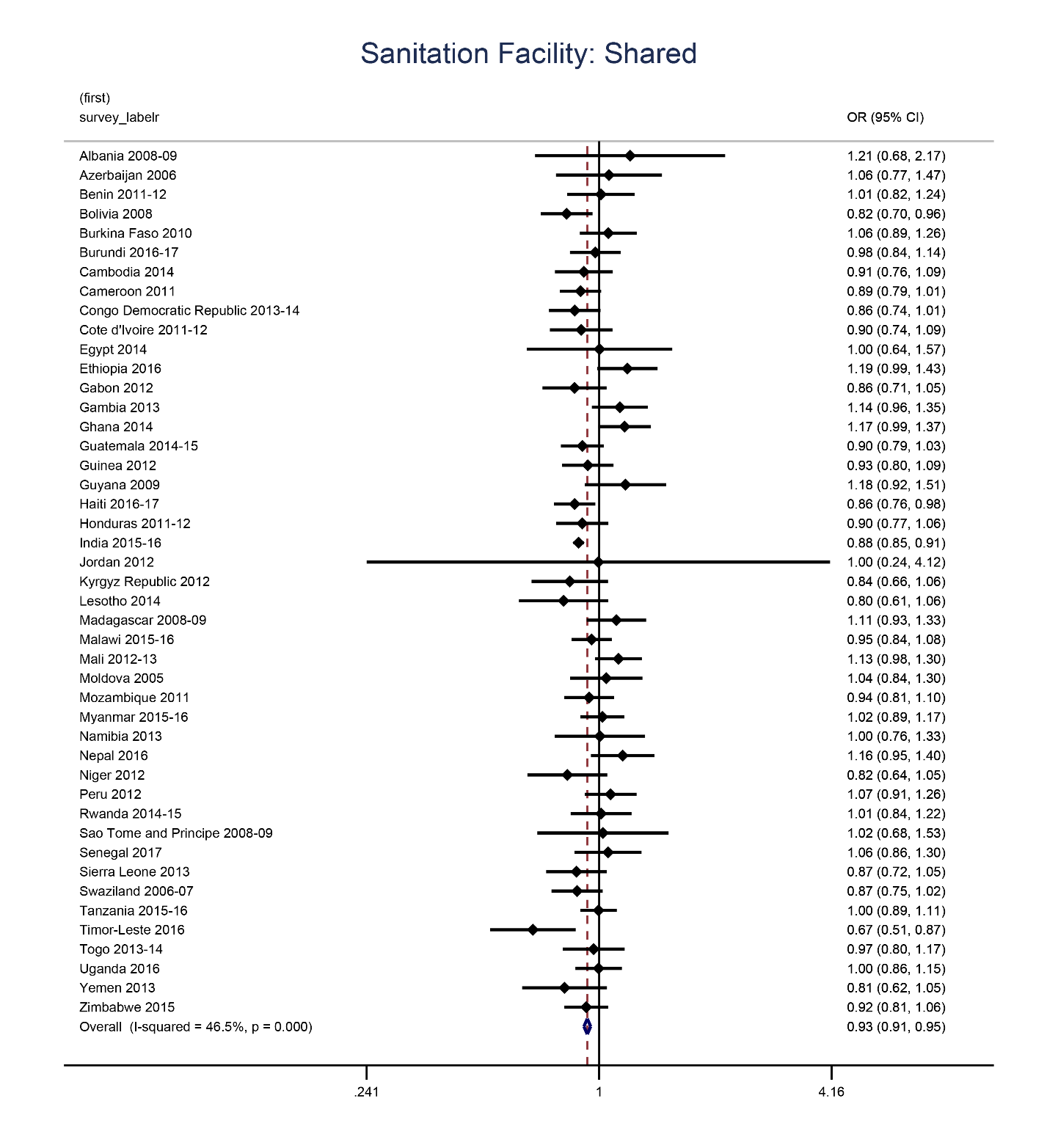


**Country**

The cumulative age adjusted OR for anemia in women exposed to a shared sanitation facility was 0. 93 (0.91-0.96), with an overall level of heterogeneity of 46.5%, p<0.001

Forest Plot 5. Association between anemia and improved and unimproved sanitation facility in women 15 to 49 years old, DHS (2006-2017)


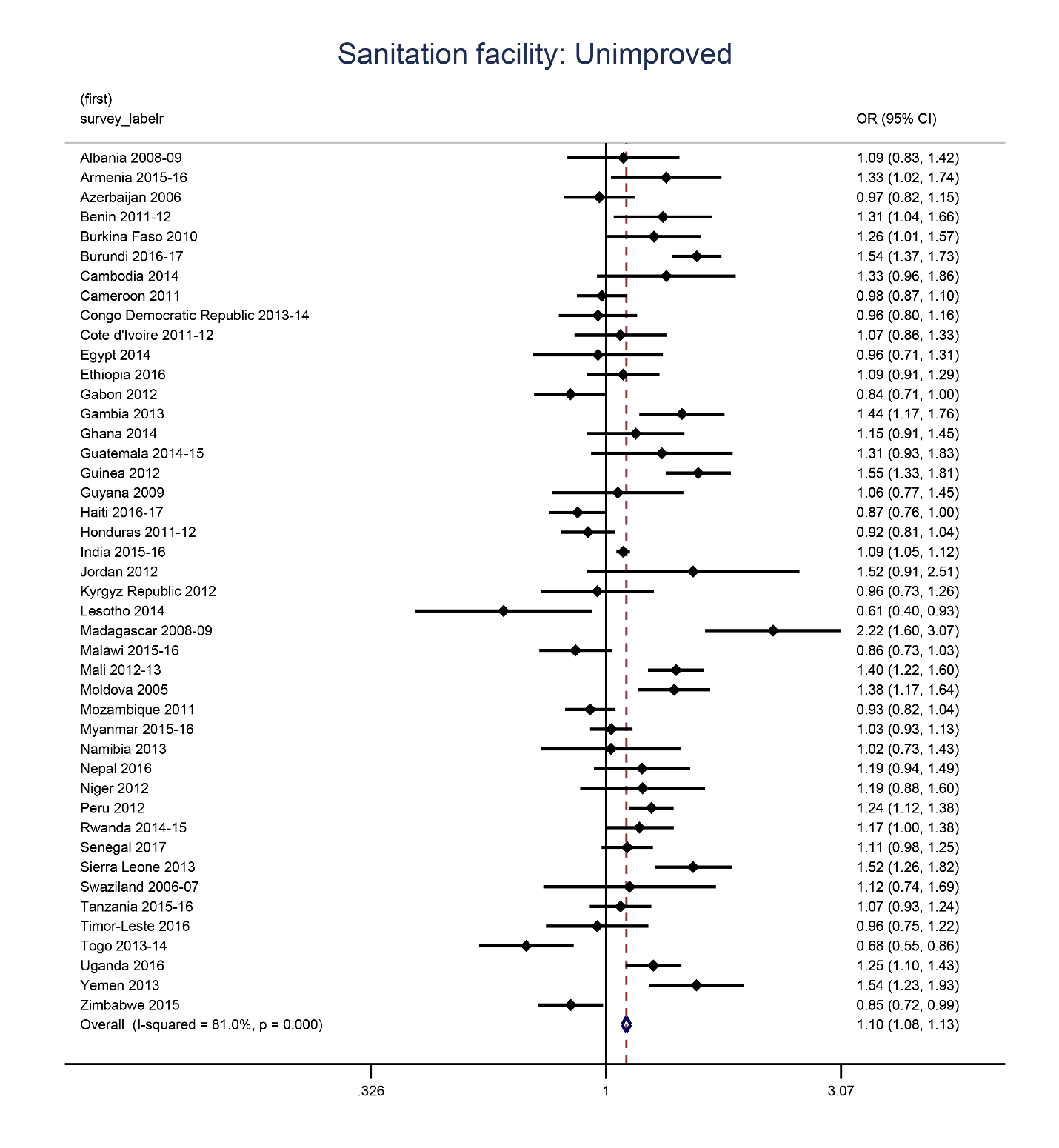


**Country**

The cumulative age adjusted OR for anemia in women exposed to a unimproved sanitation facility was 1.10 (1.08-1.13), with an overall level of heterogeneity of 81.0% p<0.001

Forest Plot 6. Association between anemia and improved and open sanitation facility in women 15 to 49 years old, DHS (2006-2017)


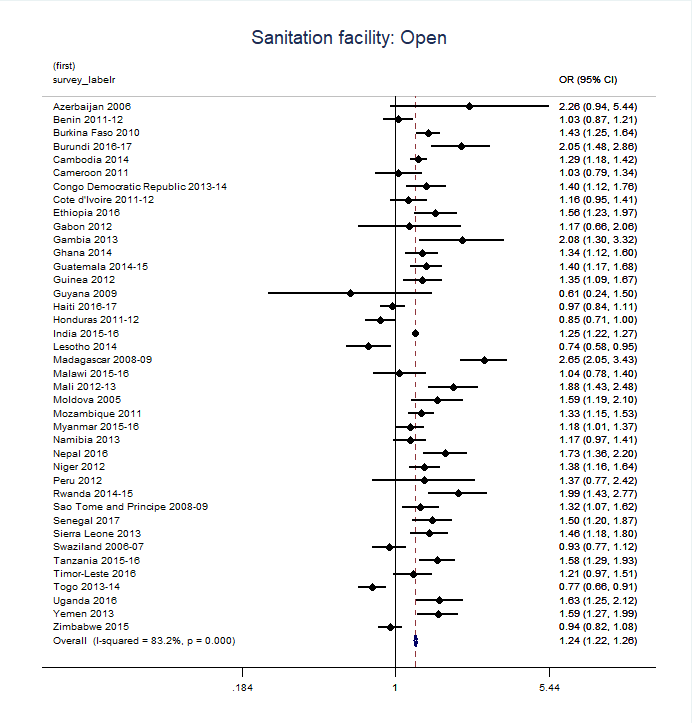


**Country**

The cumulative age adjusted OR for anemia in women exposed to an open sanitation facility was 1.24 (1.22-1.26), with an overall level of heterogeneity of 83.2%, p<0.001
